# Engineering design of platelet-mimicking therapeutic systems: multilevel biomimicry, gating strategies, and translational boundaries

**DOI:** 10.3389/fbioe.2026.1787749

**Published:** 2026-05-13

**Authors:** Jiali Chen, Xiangyu Chen, Liang Cai

**Affiliations:** 1 Department of Clinical Laboratory Medicine, Zigong Fourth People’s Hospital, Zigong, Sichuan, China; 2 Department of Transfusion Medicine, Zigong First People’s Hospital, Zigong, Sichuan, China

**Keywords:** minimal platelet interface, platelet biomimicry, platelet-mimicking nanosystems, risk-stratified gating, translational boundaries

## Abstract

Platelet-mimicking therapeutic systems have expanded well beyond hemostasis, yet clinical translation is increasingly constrained by controllability and safety-bounded deployability rather than proof-of-function. Here, we propose an engineering framework that maps design choices onto four coupled layers-structure, membrane, function, and gating-to decouple efficacy from systemic thrombotic and immunological risks. Central to this framework is a minimal platelet interface (MPI), defined as a bounded, low-gain basal state in circulation that permits lesion engagement while resisting systemic amplification. We render this interface measurable through membrane critical quality attributes (CQAs) (QC-linked membrane features), including receptor retention/orientation, shear-capture thresholds under flow, and baseline procoagulant leakage in the OFF state. We further elevate gating from simple stimulus triggering to permission-based gating (stimulus is necessary but not sufficient) by requiring risk-stratified logic, predefined activation thresholds and dynamic ranges, and validated deactivation or fail-safe constraints under worst-case conditions such as high shear and systemic inflammation. Finally, we organize applications along a risk gradient-execution for hemostasis and reversal, navigation for vascular inflammation and ischemia-reperfusion, and multilayer-gated de-risking for cancer and infection-explicitly linking biomimetic design to falsifiable safety boundaries, release criteria, chemistry, manufacturing, and controls (CMC), and regulatory requirements.

## 1 Introduction

Platelets are classically viewed as executors of hemostasis, but are increasingly recognized as dynamic, multifunctional effector cells that shape vascular repair, inflammation, immune regulation, thrombosis, and tumor-associated processes (Morrell et al., 2014; Desai et al., 2022; Scherlinger et al., 2023). Enabled by granule stores and a diverse membrane receptor repertoire, they sense injury and inflammatory cues under flow and, via cell-cell interactions and secretion, orchestrate leukocyte recruitment, endothelial responses, and coagulation-inflammation coupling (Morrell et al., 2014; Desai et al., 2022; Scherlinger et al., 2023; Zhu et al., 2024). Dysregulated platelet activation is therefore implicated in adverse outcomes across trauma hemorrhage, cardiovascular events, ischemia-reperfusion injury, infection, and cancer (Kola et al., 2021; Desai et al., 2022; Scherlinger et al., 2023; Jiang et al., 2024b).

Despite their centrality, platelet-centered interventions are bounded by hard trade-offs. Transfusion restores function but is limited by shelf life, donor dependence, immunogenicity, and storage lesions, whereas antiplatelet and anticoagulant therapies trade thrombotic protection for bleeding and still lack broadly safe, controllable reversal in several settings (Kola et al., 2021). This motivates an engineering mandate for stable, storable, and manufacturable systems that selectively reconstruct key platelet functions under verifiable control and safety-bounded deployability.

In coagulation, the cascade model describes intrinsic and extrinsic pathways converging at factor X (FX) activation to FXa, which serves as a gateway to thrombin generation. Specifically, FXa assembles with its cofactor FVa on negatively charged phospholipid membranes to form prothrombinase, converting the zymogen prothrombin (FII) into thrombin (FIIa), which then cleaves fibrinogen to fibrin to form and stabilize the clot and reinforces the cascade through feedback activation of upstream factors (Yong and Toh, 2023; Park and Park, 2024). The cell-based model emphasizes surface-organized coagulation, with initiation on tissue factor-bearing cells and amplification/propagation on activated platelet phospholipid surfaces that enable tenase/prothrombinase complex assembly (Yong and Toh, 2023). For platelet-mimicking therapeutics (PMTs), this underscores that controlling where and when a membrane becomes catalytically competent is central. Accordingly, procoagulant surface presentation should be treated as a higher-risk functional module requiring gating and bounding by measurable leakage, dynamic range (DR), and shutdown kinetics—metrics we later operationalize as critical quality attributes (CQAs) (Park and Park, 2024).

Advances in biomaterials, nanotechnology, and cell-mimicking engineering have enabled PMTs, an umbrella term for platelet-inspired nano-to microscale carriers and building blocks such as membrane-coated nanoparticles, platelet-like particles (PLPs), programmable microgels, and membrane-derived constructs (Luc et al., 2022; Safdar et al., 2024; Zhu et al., 2024; Xie et al., 2025). PMTs aim to modularize platelet functions rather than replicate whole cells, enabling programmable control of structural, biochemical, and spatiotemporal behaviors (Kunde and Wairkar, 2021; Fang et al., 2022; Han et al., 2022). We organize PMTs strategies within a four-tier structure-membrane-function-gating space. At the structure level, size, geometry, and deformability shape margination and deposition under flow (Luc et al., 2022; Jiao et al., 2023). At the membrane level, receptor repertoires support targeting, adhesion, and immune evasion (Kunde and Wairkar, 2021; Fang et al., 2022; Han et al., 2022; Safdar et al., 2024). At the function level, platforms reconstruct key outputs across distinct risk or effector modes (hemostatic, immunomodulatory, anti-infective, and tumor-interactive) (Morrell et al., 2014; Desai et al., 2022; Zhu et al., 2024; Xie et al., 2025). Finally, gating restricts activation or release to local cues that reliably mark lesion microenvironments (inflammation, acidity, shear, thrombosis) to improve selectivity and safety (Lu et al., 2019; Luc et al., 2022; Safdar et al., 2024). Accordingly, PMTs show promise in hemostasis and thrombolysis, vascular lesion targeting, sterile inflammation and infection control, and tumor delivery and immunomodulation.

Despite rapid progress, existing reviews seldom connect multi-level biomimicry to a translation-ready loop spanning manufacturability, CQAs, chemistry, manufacturing, and controls (CMC), long-term immunological safety, and regulatory constraints. This Review defines an operational minimal platelet interface (MPI), converts “platelet-likeness” into QC-ready CQAs (measurable attributes linked to release and comparability) with a minimal release-assay panel, and formalizes gain- and risk-stratified functions using gating metrics (background leakage, threshold and DR, shutdown kinetics). We further map major indications to falsifiable translational boundaries, including route and dose or exposure windows, reversibility and deactivation pathways, and CMC-driven batch consistency, within a four-layer structure-membrane-function-gating space and an efficacy-safety-translatability triangle. To improve accessibility and reduce jargon burden, Box 1 summarizes the core terminology used throughout this Review, providing operational definitions (plain-language + engineering) and representative measurable readouts.

BOX 1 **Core terminology and operational anchors used throughout this Review.**

**Platelet-mimicking therapeutics**

Engineered platelet-inspired systems designed to reproduce selected platelet-associated functions, such as lesion targeting, hemostatic support, cargo delivery, or immunomodulatory interfacing, without fully replicating native platelet biology. In this Review, platelet-mimicking therapeutics is used as an umbrella term covering membrane-coated, ligand-decorated, synthetic, and hybrid platelet-inspired platforms (Luc et al., 2022; Safdar et al., 2024; Zhu et al., 2024; Xie et al., 2025).

**Minimal platelet interface**

The minimal, bounded, low-gain platelet-inspired interface that should be selectively retained, attenuated, or gated in platelet-mimicking therapeutics to permit lesion engagement while resisting systemic amplification. Here, minimal platelet interface is used as an operational design concept rather than as a claim of whole-cell replication (Chee et al., 2024).

**Low-risk versus high-gain functional axes**

Lower-risk functions generally include margination and initial lesion capture, whereas higher-gain functions include aggregation and procoagulant amplification that may propagate systemically. This distinction helps clarify which modules should be retained, capped, gated, or excluded.

**Membrane critical quality attributes**

Operationally measurable membrane features linked to function, safety, and release readiness. Representative examples include receptor retention and orientation, ligand density, coating completeness, protein-corona susceptibility, and batch-to-batch variance (Tan et al., 2021; Fang et al., 2022; Adhalrao et al., 2024).

**Shear-capture threshold**

The minimum shear condition under which stable lesion capture occurs. A representative readout is a microfluidic adhesion curve, such as capture probability plotted against wall shear rate or shear stress (Watson et al., 2025; Zeibi Shirejini et al., 2025).

**Background leakage**

Undesired activation or off-target activity in the OFF state under trigger-free conditions. Representative readouts include basal thrombin-generation surrogates, clotting-time shifts, phosphatidylserine/Annexin V binding, complement activation, and cytokine induction (Josefsson et al., 2023; De Nardi et al., 2024; Zeibi Shirejini et al., 2025).

**Activation threshold**

The trigger level at which a locally relevant functional response first becomes detectable or enters a predefined operational window. Quantification may use trigger-titration curves across enzyme, reactive oxygen species, pH, or shear inputs.

**Dynamic range**

The separation between OFF and ON states under defined conditions. Representative quantification includes ON/OFF response ratios measured in flow-adhesion, clot-model, or release assays.

**Permission-based gating**

A stimulus is necessary but not sufficient for activation; function depends on bounded logic involving localization, dose, time window, or combinatorial inputs. This concept helps reduce false activation under challenging *in vivo* conditions (Guo et al., 2022; Sekhon et al., 2022; Zhang et al., 2025d).

**Ceiling (activity cap)**

The maximum allowable activity even in the ON state, set to prevent runaway amplification. Representative readouts include maximal thrombin generation, aggregation index, or release output under worst-case conditions (Josefsson et al., 2023; Haisma et al., 2024).

**Shutdown kinetics (τ_shutdown/stop-window)**

The rate at which activity decays after permissive cues dissipate or after a stop signal or antidote is applied. Representative readouts include deactivation half-life, microfluidic washout behavior, and antidote reversal time (Stalker, 2024; Bussin et al., 2025).

**Worst-case validation panel**

A stress-testing set intended to approximate translationally relevant systemic risk contexts. Representative scenarios include high shear, inflammation-like conditions, complement-rich media, infection-like oxidant stress, and trauma-like coagulopathy mimics (Josefsson et al., 2023; Dupuy et al., 2025; Watson et al., 2025).

## 2 Structural mimicry: size, shape, and mechanics

From a hemodynamic-constraint perspective, structural mimicry is defined by three coupled variables, size, shape, and mechanical compliance (Sugihara-Seki and Takinouchi, 2021; Ashrafmansouri et al., 2024). Size governs near-wall enrichment and margination; shape modulates rolling and adhesion contact geometry; and compliance controls deformation, microcirculatory passage, and phagocytic clearance. Together, they determine deposition efficiency in whole blood and set an upper bound on the safety window. Structural mimicry should therefore be framed as constraint matching under blood rheology rather than geometric resemblance alone.

### 2.1 Size (near-wall enrichment window)

**FIGURE 1:**
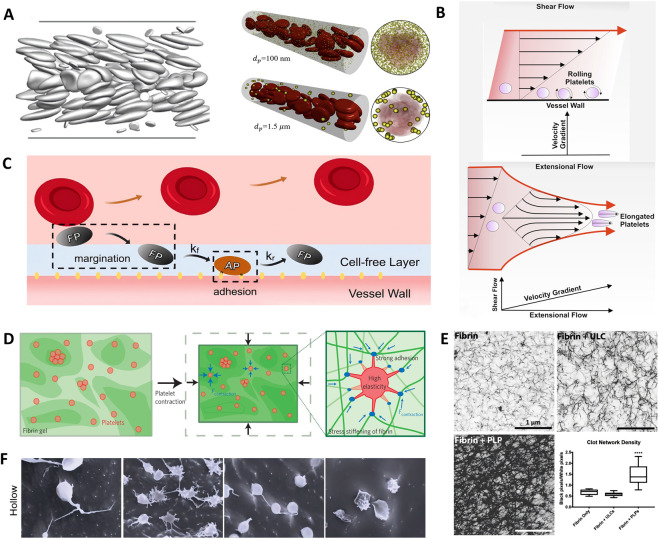
Hemodynamics-constrained structural biomimicry of platelets: size, shape, and mechanical compliance. **(A)** Hemodynamics-driven size selection for near-wall accumulation (Tuna et al., 2024). Copyright 2024, Int J Mol Sci. **(B)** Shear and extensional flows impose distinct near-wall force fields that govern platelet rolling and deformation (Tuna et al., 2024). Copyright 2024, Int J Mol Sci. **(C)** Shear-dependent near-wall states of platelet margination (Li L. et al., 2023). Copyright 2022, Elsevier. **(D)** Mechanical compliance and active contraction as design benchmarks for platelet-inspired structures (Lam et al., 2011). Copyright 2011, Springer Nature. **(E)** Representative experimental evidence for fibrin network densification by anchored, compliant platelet mimetics (Nandi et al., 2019). Copyright 2019, Royal Society of Chemistry. **(F)** Structural evolution enables programmable mechanical behavior in platelet-mimetic microgels (Sproul et al., 2018). Copyright 2018, Adv Biosyst.

Vascular wall access first depends on whether carriers can enter the near-wall region in whole blood, where transport is governed by red blood cell (RBC)-driven margination and the near-wall cell-free layer (CFL) (Tuna et al., 2024). Simulations and microfluidic studies suggest that submicron nanoparticles often remain in the RBC-rich core and fail to sustain near-wall enrichment, whereas natural platelets (≈2–3 μm) access the CFL more effectively via persistent RBC-platelet collisions (Cooley et al., 2018; Tuna et al., 2024). Thus, platelet-inspired design is not simply “smaller is better”; the micron scale represents a hemodynamically selected window for near-wall access (Figure 1A).

### 2.2 Shape (contact dynamics and adhesion probability)

Within the micron-scale access window, non-sphericity increases wall-contact likelihood. The flattened discoid shape of resting platelets promotes tumbling and rolling with lateral drift under shear, increasing transient near-wall encounters with exposed adhesive ligands such as von Willebrand factor (vWF) (Cooley et al., 2018; Tuna et al., 2024). This advantage is not explained by surface area alone: even when total ligand number is fixed and ligand density is therefore lower on non-spherical surfaces, discoid microparticles can outperform spherical controls in adhesion and retention (Cooley et al., 2018) (Figure 1B). However, the benefit is shear-bounded. Dissipative particle dynamics simulations suggest that at moderate shear, RBC-platelet collisions support relatively stable CFL enrichment, whereas at higher shear, wall-induced lift and lateral velocity fluctuations bias platelets toward a near-CFL state with unstable contact (Li L. et al., 2023). Thus, structural parameters should be tuned to the shear environment of the target vascular bed rather than assuming that “platelet-like shape” universally yields effective wall interactions (Figure 1C).

### 2.3 Mechanical compliance (deformation, anchoring, and network integration)

Compliance sets the mechanical dimension of structural mimicry. Single-cell force spectroscopy suggests activated platelets have an effective elastic modulus of roughly 10 kPa and remain highly deformable, while actin-myosin contraction generates forces in the tens of nanonewtons that compact fibrin, stiffen clots, and drive mechanobiological feedback (Lam et al., 2011) (Figure 1D). These values provide practical benchmarks for synthetic platelet analogues. Critically, performance depends on softness coupled to spatial anchoring: low-crosslinking polymer microgels can deform and spread under confinement and, when fibrin-anchored via specific ligands, induce local densification and improve bulk mechanics (Nandi et al., 2019; Nandi et al., 2020b). Soft particles without anchoring may not reinforce comparably and can perturb network architecture (Figure 1E). Degradable cores or hollow microgels further introduce time-dependent structural evolution as a temporal control axis for programmable mechanics (Sproul et al., 2018) (Figure 1F).

From a broader nanomedicine perspective, the CFL-accessible size window, together with deposition and clearance advantages from non-sphericity and deformability, provides a general physical basis for platelet-inspired structural design (Anselmo et al., 2014; Key et al., 2015; Palange et al., 2017). However, not all platforms need to fully recapitulate platelet hemodynamics; some are better positioned as multifunctional hemostatic carriers that prioritize size and compliance while only partially adopting platelet-like transport behaviors (Shuai et al., 2025). More fundamentally, platelet biomimicry translates principles of flow physics into the boundary conditions that govern hemostasis and thrombosis, where multiscale mechanosensitive coupling becomes decisive. Specifically, vWF’s shear-dependent mechanics governs the capture regime, force-dependent bond lifetimes shape rolling-to-arrest transitions, and nonlinear fibrin mechanics dictates how particle compliance translates into clot reinforcement. (Hadji and Bouchemal, 2022; Risser et al., 2022).

In summary, size governs near-wall entry, shape modulates contact dynamics, and compliance enables network integration and mechanical contribution. Structural mimicry is therefore a window-matching problem rather than a single-point optimization. For a given vascular bed, designs should co-tune size, geometry, and deformability to the local shear environment and task demands. The goal is to maximize near-wall residence while preserving non-occlusive clearance, so that structural gains translate into controllable functional outputs (Lam et al., 2011; Cooley et al., 2018; Sproul et al., 2018; Nandi et al., 2019; Nandi et al., 2020b; Li L. et al., 2023; Tuna et al., 2024).

## 3 Membrane mimicry: interface engineering

The MPI guides membrane-layer engineering. For a given indication, low-risk axes for navigation, initial adhesion, and immune entry can be preferentially retained, whereas high-risk axes linked to aggregation and procoagulant amplification should be attenuated or permissioned via gating. Accordingly, membrane mimicry should be judged by engineering deliverables rather than appearance, translating “platelet-likeness” into quantifiable membrane CQAs and a minimal release package that enables downstream gating and indication-specific translational boundaries (Fang et al., 2022). While MPI is a useful conceptual scaffold, its design and safety value depends on stating where functional coupling concentrates and how high-gain transitions are constrained. Here, we provide a mechanistic specification of MPI by identifying risk-bearing coupling nodes that connect early lesion engagement to aggregation, immune amplification, and procoagulant conversion, and by formulating “retain-attenuate-gate” rules paired with measurable boundary conditions for characterization and worst-case stress testing.

### 3.1 Mechanistic specification of the MPI

Platelet-inspired systems must contend with the fact that functions often treated as separable modules, including near-wall transport and capture, stable adhesion, aggregation, immune recruitment, and procoagulant conversion, are mechanistically coupled (Girish et al., 2020; Nellenbach and Brown, 2022). In native platelets, early capture mediated by the glycoprotein Ib alpha (GPIbα)-vWF interaction under shear can propagate into integrin activation and aggregation (Yang H. et al., 2020; Zou et al., 2022). Immune-facing ligands can engage thromboinflammatory feedback, and phosphatidylserine (PS) exposure links coagulation amplification to inflammatory signaling (Engelmann and Massberg, 2013; Iba and Levy, 2018). Accordingly, we define the MPI as an engineering specification rather than a static receptor list. The interface should maintain a low-gain basal state in circulation that supports navigation and early lesion recognition, while entry into high-gain amplification programs is permissioned and confined to a bounded state window (Chee et al., 2024; Li et al., 2024d).

**FIGURE 2:**
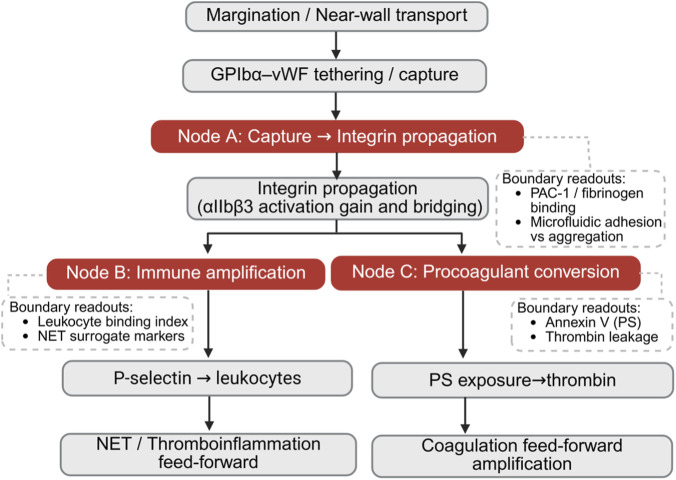
MPI mechanistic specification: coupling nodes and boundary readouts. Platelet-inspired functions are mechanistically coupled from near-wall transport and shear-mediated capture to integrin propagation, procoagulant conversion, and immune amplification. Three coupling nodes (A–C) mark risk-bearing transitions, and representative boundary readouts are shown for each node to operationally bound and verify high-gain propagation in MPI-oriented designs. (Created with www.biorender.com).

To make MPI actionable, we map the major coupling nodes that convert beneficial lesion engagement into systemic risk (Figure 2), reflecting the coupled nature of platelet adhesion, aggregation, procoagulant amplification, and immune interactions (Girish et al., 2020; Nellenbach and Brown, 2022) and the need to bound high-gain transitions through permissioned gating (Chee et al., 2024; Li et al., 2024d). One coupling node is the transition from shear-mediated tethering to integrin-driven propagation, in which capture events propagate into integrin αIIbβ3 (also known as CD41/CD61) activation and bridging (Yang H. et al., 2020; Zou et al., 2022). This transition governs whether transient contact becomes rapid thrombus growth; engineering levers include limiting agonism/avidity at capture and constraining propagation to integrin activation. A second coupling node is the immune-thrombotic amplification axis, for example P-selectin mediated leukocyte engagement with downstream neutrophil activation and NET-associated feedback, which can increase thrombosis burden under inflammatory milieus (Engelmann and Massberg, 2013; Iba and Levy, 2018). Thus, immune-facing engagement should be explicitly bounded to avoid feed-forward immunothrombosis. A third coupling node is procoagulant conversion through PS exposure, which provides a catalytic surface for tenase and prothrombinase assembly and can markedly elevate thrombin generation (Sekhon et al., 2022; Yong and Toh, 2023; Park and Park, 2024). This motivates default-off procoagulant presentation with permissioned unmasking and a defined shutdown route. In the MPI framework, these coupling nodes are treated as risk-bearing transitions that must be attenuated or gated by design.

We therefore formulate MPI as a retain-attenuate-gate prescription (Fang et al., 2022). Features that primarily enable navigation and early lesion recognition are retained, such as margination-promoting mechanics and shear-assisted, low-agonism tethering (Cooley et al., 2018; Tuna et al., 2024). High-gain aggregation is attenuated by limiting propagation from capture to integrin activation, for example by constraining ligand density and avidity, using low-agonism adhesive presentation, or controlling the conformation of integrin-mimetic elements (Desai et al., 2022; Sharifi-Rad et al., 2023; Schorr et al., 2025). Highest-risk amplification programs, particularly PS exposure and strong immune amplification, are implemented as default-off capabilities that require localized permission cues, and must include predefined termination/shutdown behavior under loss-of-control scenarios (Sekhon et al., 2022; Chee et al., 2024; Li et al., 2024d). As summarized in Figure 2, each coupling node is paired with representative boundary readouts for engineering verification. These include readouts of integrin activation state and bridging propensity, PS exposure and baseline procoagulant leakage, thrombin generation surrogates, and immune-binding amplification markers (Yong and Toh, 2023; Park and Park, 2024). This links the conceptual MPI design space to verifiable characterization, release-oriented testing, and worst-case stress evaluation.

### 3.2 Paradigms of membrane mimicry: from transfer to engineering

**FIGURE 3:**
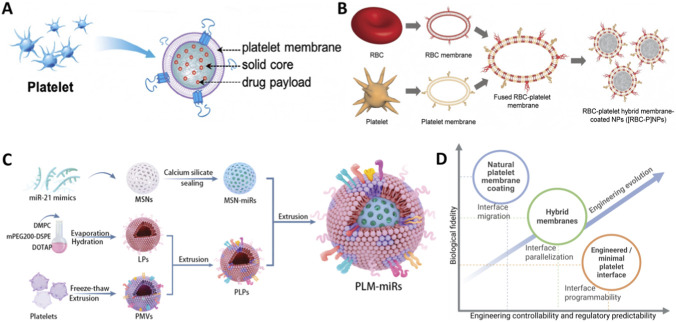
Engineering paradigms and trade-offs in platelet biomimetic interface design. **(A)** Natural platelet membrane coating as an interface migration paradigm (Wang et al., 2020a). Copyright 2020, Small Struct. **(B)** Hybrid membranes as an interface parallelization paradigm (Dehaini et al., 2017). Copyright 2017, Adv Mater. **(C)** Engineered membranes enable programmable and MPIs through interface rewriting (Tan et al., 2021). Copyright 2021, Adv Sci. **(D)** Engineering trade-offs among platelet biomimetic interface strategies.

Membrane mimicry is a hallmark top-down route in platelet biomimicry. Instead of bottom-up decorating synthetic surfaces with a few ligands, platelet membrane coating transfers the native membrane as a system-level interface onto nanoparticles, inheriting coupled receptor-network behaviors (Wang et al., 2020a). Platelet membranes organize cooperative adhesion and immune axes, including glycoprotein (GP)Ib/IX/V, GPVI, and integrin αIIbβ3 as well as CD47 and P-selectin (CD62P) (Jiang et al., 2020; Wang et al., 2020a). In concert with shear, exposed lesion extracellular matrix (ECM), and endothelial activation, these networks implement dynamic recognition logic, such as vWF-dominant capture under high shear versus stronger collagen or fibrin engagement under lower shear or tissue-injury contexts, producing a multi-tier adhesion hierarchy (Wang et al., 2020a). Thus, native coating transfers interface recognition logic rather than simply stacking targeting tags (Figure 3A). Native membrane coating typically uses freeze-thaw vesiculation followed by sonication or extrusion fusion onto nanoparticle cores, producing reproducible core-shell structures with about 10 nm membrane thickness. Vesicle-like shifts in size and zeta-potential indicate interfacial reconstruction rather than adsorption (Jiang et al., 2020). The intent is to retain the membrane interface while stripping cytosolic and granule contents, preserving adhesion and immunomodulatory proteins without introducing procoagulant drive. Accordingly, evaluation should prioritize receptor-network retention and effective exclusion of pro-reactive contents and procoagulant cues, not merely the presence of a coating.

Native coating credibility should rest on an engineerable evidence chain that combining proteomic and immunoreactivity consistency with mechanistically falsifiable tests. For example, retention of CD47, CD41, and P-selectin with preserved antibody binding supports epitope availability after coating (Jiang et al., 2020). Conversely, the loss of targeting advantage in platelet-poor plasma from aspirin and ticagrelor treated patients argues for receptor-mediated targeting over passive retention (Hua et al., 2024). Functionally, platelet membranes implement cooperative, multi-cue recognition across activated endothelium, exposed ECM, and fibrin networks, improving selectivity for pathological interfaces in atherosclerosis or thromboinflammation contexts (Wei et al., 2018; Wang et al., 2020b; Liu B. et al., 2023). However, certain axes can introduce positive feedback, such as P-selectin driven platelet-neutrophil interactions promoting NETs that further increase particle capture (Xiao et al., 2024). Therefore, enrichment mechanisms should be explicitly bounded rather than treated as inherently safe.

Beyond adhesion-based recognition, CD47-mediated self signaling suppresses phagocytic clearance, prolongs circulation, and reduces nonspecific liver and spleen uptake (Jiang et al., 2020; Wang et al., 2020a; Xiao et al., 2024). In inflammatory settings, platelet membranes can also enrich lesions through P-selectin-P-selectin glycoprotein ligand-1 (PSGL-1) interactions interactions and immune-cell co-migration; together with CD47, this supports an interface strategy combining immune-cell hitchhiking with immune evasion, reducing reliance on vessel-wall recognition or enhanced permeability and retention and leveraging immune-cell networks for active transport (Lin et al., 2025). Across organs and disease models, recurrent lesion enrichment coupled with membrane-protein retention suggests that coated systems function as transferable interface-logic modules rather than disease-specific solutions (Dong et al., 2023; Fei et al., 2024).

Most studies have not systematically compared resting versus activated platelet membranes in receptor repertoire, orientation, or epitope availability, so the field understands retention and performance better than how membrane state can be used to generate controllable functional differences (Wei et al., 2018; Jiang et al., 2020; Wang et al., 2020b; Chen et al., 2025c; Lin et al., 2025). Two engineering routes follow. Hybrid membranes fuse sources to redistribute functions and combine recognition and *in vivo* behaviors, whereas engineered membranes rewrite parameters via insertion, reconstruction, or replacement to tune receptor density, orientation, and responsiveness for decoupling and manufacturability (Zhang et al., 2018; Safdar et al., 2024; Liu B. et al., 2025). The engineering gradient and key trade-offs among membrane-biomimetic paradigms are summarized in Table 1.

**TABLE 1:** Engineering gradient of platelet membrane biomimetic strategies.

Strategy	Engineering positioning	Inheritable capability	Dominant risk sources	Minimal falsifiable validation set	Translational CQAs
Natural platelet membrane coating	Interface logic transfer (fidelity-dominant)	Full receptor repertoire; multi-axis recognition	Procoagulant amplification (αIIbβ3); P-selectin-NETs positive feedback; membrane state uncertainty	True membrane fusion; receptor retention with conformational availability; exclusion of platelet activation	Receptor density/orientation; membrane state consistency; coagulation/immune safety boundaries
Hybrid membranes	Functional redistribution (navigation-effector decoupling)	Platelet targeting + complementary *in vivo* behavior	Interface composition drift; scale-up-induced functional deviation	Membrane-level fusion; controllable fusion ratio; batch stability	Fusion ratio window; batch consistency; scale-up robustness
Engineered membranes	Programmable interface reconstruction (spec-driven)	High controllability and manufacturability	Reduced biological fidelity; insufficient pathological selectivity	Receptor density/orientation windows; shear-responsive behavior; explicit safety limits	Explicit interface specs; surrogate equivalence metrics; scalability and regulatory feasibility

Abbreviations: αIIbβ3, integrin alpha IIb, beta 3; NETs, neutrophil extracellular traps; GPIbα, glycoprotein Ib alpha; vWF, von Willebrand factor; CD47, cluster of differentiation 47; QC, quality control; DR, dynamic range; τ, time constant.

For most indications, native membranes provide an upper-bound reference for receptor repertoire and interfacial fidelity. Under state targeting, low-risk modules for navigation and initial adhesion (e.g., GPIbα-vWF capture and collagen-binding) can be preferentially retained. Immune entry or evasion modules (e.g., CD47 and selected P-selectin associated interactions) can also be preserved when needed. In contrast, high-risk amplification modules (e.g., αIIbβ3 driven aggregation and potentially procoagulant interfaces) should be explicitly identified and evaluated for systemic spillover risk (Kunde and Wairkar, 2021). Accordingly, QC should prioritize retention and conformational availability of key receptors, membrane-state-driven repertoire differences, and bounded perturbations in platelet activation and coagulation readouts (Liu H. et al., 2023). Here, “ceiling” denotes an absolute upper bound for a given readout, whereas “Δ-bound” denotes a bounded deviation relative to a reference condition (e.g., a vehicle control, a blank carrier control, or a reference batch/material). “Threshold + DR” defines an operational activation/capture range (threshold plus dynamic range), and “τ(time constant)” specifies the required termination/shutdown time constant.

Hybrid membranes fuse a platelet-recognition interface with *in vivo* behavioral advantages from other membrane sources (Figure 3B). Fuorescence resonance energy transfer and co-localization support a unified interface that co-displays RBC and platelet molecules while retaining CD47-mediated immune evasion, coupling long circulation and anti-phagocytosis with injury recognition and adhesion on the same surface (Dehaini et al., 2017; Yang M. Y. et al., 2023; Li et al., 2024e). Many designs confine platelet-associated functions to positioning and adhesion, reducing the likelihood of prothrombotic amplification (Tan et al., 2021; Yang M. Y. et al., 2023; Li et al., 2024e). From a manufacturing perspective, an extract-then-fuse workflow enables ratio quantification and improves batch consistency (Adhalrao et al., 2024). Hybrids that combine platelet membranes with immune-cell extracellular vesicles (EVs) or exosomes further implement division of labor, with platelet membranes providing navigation and *in vivo* behavior while EV cargo supplies effector output, reducing systemic risks associated with copying hemostatic functions (Li et al., 2024d; Xie et al., 2024; Lu et al., 2025). Thus, hybrid membranes align with the MPI by concentrating platelet contributions on navigation and adhesion (e.g., GPIbα-vWF for high-shear capture and CD47 for immune evasion) while avoiding or amplitude-limiting aggregation and procoagulant amplification. QC should verify genuine fusion, controllable composition ratios, and quantifiable batch drift to prevent composition and functional drift during scale-up.

Engineered membranes make platelet interfaces parameterizable. By anchoring or reconstructing receptors, chemically modifying membranes, or partially replacing them with synthetic components, designs move from natural inheritance to task-configured interfaces (Tan et al., 2021; Safdar et al., 2024; Liu B. et al., 2025) (Figure 3C). The central value is decoupling physiological stability and low nonspecific interactions from lesion-localized interface transitions that restrict activity in space and time (Liu B. et al., 2025). Reassembly with fusogenic liposomes can split navigation from entry, letting platelet membranes set targeting while synthetic modules control cellular access (Tan et al., 2021), and platelet-like synthetic liposomes offer definable composition, batch consistency, and scalability for CQAs-based scale-up (Wang et al., 2026). In this paradigm, the MPI becomes a set of tunable specifications, including retained navigation or immune-entry axes, removed or weakened aggregation and procoagulant axes, and adjustable receptor density or orientation, shear-switch thresholds, and termination conditions. The key falsifiable question is whether selective pathological recognition can be maintained after simplifying interfaces while keeping systemic thrombotic and immunological risks within releasable bounds.

Finally, evaluation should move from platelet-likeness to satisfaction of engineering constraints. Key metrics include proof of membrane-level fusion rather than physical mixing, retention and correct orientation of key proteins, batch-consistent membrane ratios and proteomic profiles, and confinement of function to recognition and navigation without systemic hemostatic perturbation (Dehaini et al., 2017; Tan et al., 2021; Yang M. Y. et al., 2023; Adhalrao et al., 2024; Li et al., 2024e). In this view, native membranes set an upper bound for receptor repertoire and fidelity, whereas hybrid and engineered membranes provide tunability, manufacturability, and functional decoupling, advancing membrane mimicry from interface transfer to programmable interface engineering (Zhang et al., 2018; Safdar et al., 2024; Liu B. et al., 2025) (Figure 3D).

### 3.3 Lesion recognition: adhesion and immunity

**FIGURE 4:**
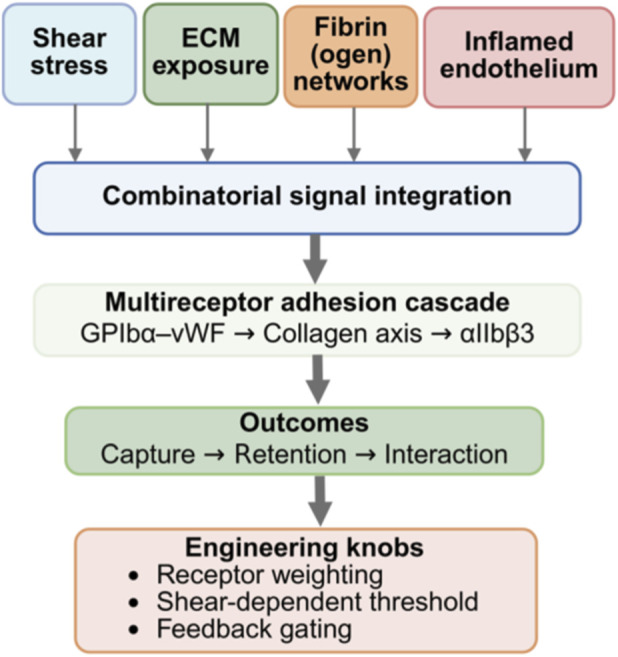
Combinatorial state recognition and adhesion-immune coupling by platelet-mimetic interfaces. Platelet-mimetic interfaces achieve lesion enrichment by integrating hemodynamic, matrix, fibrin, and inflammatory cues into multireceptor adhesion cascades, enabling state-level recognition rather than single-target binding. Engineering control over receptor composition, shear-dependent switching, and feedback strength governs targeting robustness and safety boundaries. (Created with www.biorender.com).

Platelet membrane-mimetic nanomaterials enrich at lesions across thrombotic, inflammatory, ischemic, and tumor settings. Their advantage is not single-marker targeting but partial reconstitution of a multivalent, hierarchical, mechanosensitive platelet interface that integrates shear, matrix exposure (collagen or vWF), fibrin networks, endothelial activation, and immune recruitment into capture and residence behaviors (Fang et al., 2022; Safdar et al., 2024). Accordingly, the effective target is the pathological state rather than an isolated marker (Figure 4).

This combinatorial state recognition is expressed as a cooperative multi-receptor adhesion cascade. Under high shear, platelets progress from transient contact to firm retention via GPIbα-vWF initial capture, collagen-binding deceleration and stabilization, and αIIbβ3 engagement with fibrin or fibrinogen for final anchoring. Platelet membrane coating can inherit this multi-axis unit, allowing biomimetic systems to shift dominant binding pathways with changing shear and sustain near-wall residence (Yang H. et al., 2020). The cascade also offers an engineering handle for functional decoupling: when capture or navigation is desired without amplification, retaining GPIbα-vWF while attenuating aggregation-associated modules can reduce systemic prothrombotic risk (Yang H. et al., 2020; Zou et al., 2022). The relative weights of combinatorial cues vary across diseases and thus determine the optimal interface configuration. Atherosclerotic plaques combine endothelial activation with ECM exposure, so membrane-mimetic systems often enrich in inflamed or structurally unstable regions and can respond even to early endothelial activation (Wei et al., 2018). In acute ischemic stroke, thrombo-inflammation is more tightly coupled, producing a triad of thrombus or fibrin networks, inflamed endothelium, and immune recruitment (Li M. et al., 2025). Accordingly, the engineering objective shifts from maximizing adhesion strength to maximizing selective responsiveness to cue combinations, motivating receptor-combination redesign and reweighting in hybrid and engineered membranes (Wei et al., 2018; Zou et al., 2022; Li et al., 2024c; Li M. et al., 2025).

Beyond static recognition, membrane-mimetic enrichment can couple to pathological evolution and become self-reinforcing. Vascular disruption exposes collagen and recruits platelets, and biomimetic particles retaining adhesion modules can be co-recruited to create local positive feedback; in tumor vascular targeting, damage-induced microthrombosis and matrix exposure can similarly amplify recruitment signals over time (Li et al., 2021; Ding et al., 2023). While such feedback can extend residence, it may also amplify thrombosis or inflammatory cascades, so designs must predefine whether amplification is intended and, if so, specify thresholds and termination conditions that interface with downstream gating and reversal strategies (Li et al., 2021; Ding et al., 2023). In parallel, platelet-mimetic interfaces can engage immune networks through cell-cell interactions. This can create a continuous chain of localization, interaction, and modulation *in vivo*. In tumor and inflammatory settings, these interfaces may reshape immune-cell interaction axes and amplification loops, with the CD47-signal regulatory protein alpha axis contributing to both immune evasion and immune reprogramming (Gao et al., 2022; Li F. et al., 2022; Li X. et al., 2025; Zhang Y. et al., 2025). Not all applications require a full platelet membrane. Receptor-tunable platelet micro-ghosts suggest that initial adhesion is often dominated by the GPIbα-vWF axis; selectively retaining it enables recognition of aberrant flow and vWF-enriched regions, whereas attenuating aggregation-related receptors such as αIIbβ3 can reduce nonspecific aggregation and prothrombotic risk (Zou et al., 2022). This supports modular inheritance as a membrane-engineering principle and raises a translational question: can a standardized MPI preserve state targeting while maximizing the safety window and improving QC controllability (Fang et al., 2022; Zou et al., 2022). From an engineering perspective, focus shifts from single-target hits to three tunable parameters: receptor ratios for combinatorial cues, shear-switching thresholds in the adhesion cascade, and termination conditions for amplification and immune interactions. These variables jointly govern enrichment robustness and risk spillover, shaping gating design and CQAs definitions, including receptor repertoire, shear-dependent adhesion, and platelet activation or coagulation bounds.

## 4 Functional reconstruction: modules and the safety layer

At the functional level, the goal is not to replicate native platelet behaviors as a whole, but to identify and modularly reconstruct decisive functional nodes in hemostasis and related pathologies. Adhesion, activation-aggregation, procoagulant amplification, and immune interactions are coupled along the same time axis; indiscriminate “full-function biomimicry” imports both hemostatic benefits and systemic prothrombotic risks, narrowing the safety window and translational potential (Girish et al., 2020; Nellenbach and Brown, 2022). A more viable approach is to selectively inherit key functional axes while placing high-gain loops under verifiable safety-layer constraints, such as capped background leakage, lesion-specific triggering, and demonstrable termination or deactivation with bounded systemic perturbation. Additionally, three often conflated strategies should be distinguished: artificial platelets that reconstruct function, coagulation-factor delivery systems using activated platelets as triggers, and platelet-membrane-mimetic strategies that primarily optimize drug distribution. Their clinical positioning, safety liabilities, and translational pathways differ (Wang et al., 2020b; Girish et al., 2022).

**FIGURE 5:**
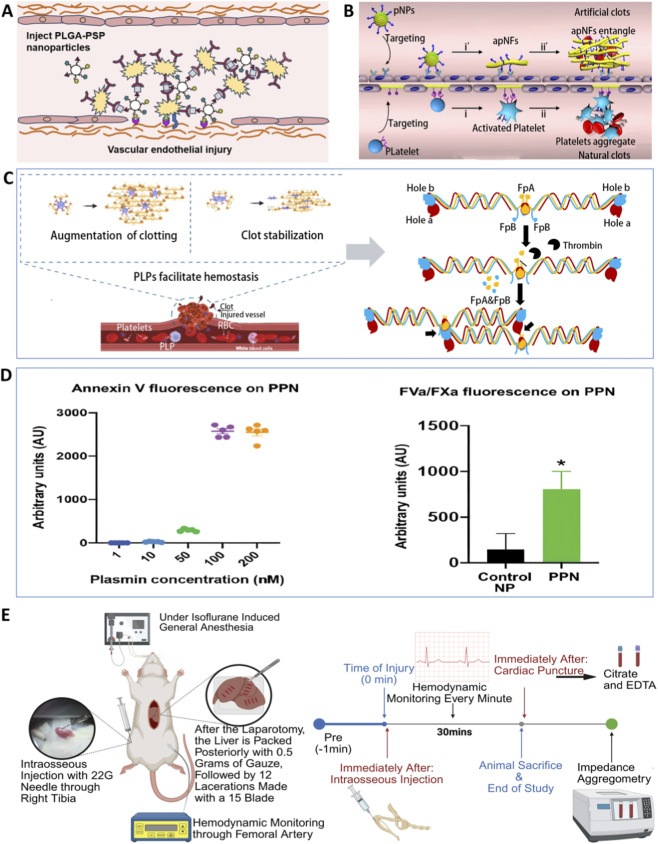
Modular reconstruction of platelet-inspired hemostatic functions with decoupled efficacy and safety. **(A)** Adhesion-aggregation module enabling injury-site localized hemostatic assembly without systemic platelet activation (Fu et al., 2025). Copyright 2025, Int J Nanomedicine. **(B)** Self-amplifying activation-aggregation with inherent thrombotic risk (requires gating) (Yang P. P. et al., 2020). Copyright 2020, Sci Adv. **(C)** Mechanical modules for clot densification and stabilization without amplification of coagulation kinetics (Nandi et al., 2021; Nellenbach et al., 2024). Copyright 2021, Adv Ther; Copyright 2024, Sci Transl Med. **(D)** Constrained procoagulant amplification via PS exposure (Sekhon et al., 2022). Copyright 2022, Sci Transl Med. **(E)** Translational deployability of synthetic platelet technologies (Liu Z. et al., 2025). Copyright 2025, J Thromb Haemost.

Adhesion-aggregation modules: localization and local construction. This module class aims to assemble hemostatic function at the lesion interface rather than inducing systemic activation. A multi-ligand poly (lactic-co-glycolic acid) bearing platelet-mimetic surface peptides (PLGA-PSP) integrates collagen, vWF, and fibrinogen-mimetic peptides to drive stepwise aggregation from initial positioning to recruitment of circulating platelets, enhancing local hemostasis without directly triggering systemic platelet activation (Fu et al., 2025) (Figure 5A). Representative chemical structures of the peptide-based recognition segments discussed here, together with typical conjugation chemistries used for surface presentation, are summarized in Supplementary Figure S1. Liposome-based synthetic platelets integrate vWF/collagen binding with a GPIIb/IIIa-fibrinogen-mimetic axis and, in large-animal trauma models, reduce blood loss and improve hemodynamics while multiple systemic readouts show no abnormal thrombosis (Srinivasan et al., 2024). Together, these results support a safety-layer principle: anchor effector assembly to the injury interface to preserve a deployable safety window. Indication-specific “module prescriptions” are essential because missing links differ across bleeding etiologies. Across von Willebrand disease (VWD) subtype models, Roullet et al. showed that VWD-2B requires collagen binding as a prerequisite that must synergize with aggregation modules, whereas in vWF-deficient models near-wild-type performance emerges only when collagen binding, vWF-related adhesion, and aggregation modules are combined, yielding thrombus spatial distributions closer to wild type (Roullet et al., 2023). Thus, module designs should target etiology-specific gaps rather than simply stacking elements.

Activation-aggregation cascade reconstruction: feasible but requires gating. Materialized reconstruction of activation/aggregation dynamics is possible, but self-amplifying loops must be explicitly placed under gating. Peptide self-assembled nanoparticle(s) (pNPs), upon binding CD105, transform from particles into nanofibers and expose self-recognition sequences, continuously recruiting additional pNPs and forming a self-amplifying assembly (Yang P. P. et al., 2020) (Figure 5B). This demonstrates engineered dynamic aggregation, but it depends on strictly lesion-specific triggering and a verifiable termination or deactivation mechanism; otherwise, such designs should be viewed as proof-of-concept rather than a default enhancement route (Yang P. P. et al., 2020).

Mechanical-function modules: clot densification, stabilization, and repair-friendly microenvironments. Mechanical modules can partially reduce reliance on procoagulant amplification by densifying and stabilizing clots while shaping a repair-favorable microenvironment. Ultra-soft platelet-like microgels anchor to fibrin, spread via high deformability, and impose strain that collapses networks and drives clot contraction, reducing blood loss and promoting healing without off-target coagulation (Nellenbach et al., 2024). The fibrin-affine microgels selectively mimics knob B-hole b interactions to increase fiber density and antifibrinolytic stability while minimizing disruption of dominant physiological fibrin polymerization kinetics, illustrating synergy between target selection and mechanical behavior (Nandi et al., 2021) (Figure 5C). Beyond hemostasis, PLPs have been reported to promote fibroblast migration and regenerative indices. In traumatic brain injury, to correct early hypocoagulability while attenuating subacute neuroinflammation, suggesting that stabilization of the hemostatic microenvironment and its downstream inflammation and repair cascades should be treated as an evaluation dimension for functional biomimicry (Nandi et al., 2019; Todd et al., 2021).

Procoagulant amplification modules: reachable only through strict gating. Procoagulant amplification is feasible but requires strict gating because PS exposure both enables coagulation-factor complex assembly and drives systemic thrombotic risk. Injury-responsive PPNs shield PS in circulation and expose it only in bleeding microenvironments with high fibrinolytic activity, achieving spatially restricted amplification and improving survival without evident systemic thrombosis (Sekhon et al., 2022) (Figure 5D). Conversely, PS blockade on activated platelets or damaged endothelium can delay arterial thrombotic occlusion without markedly prolonging hemostasis time, suggesting partial decoupling of procoagulant modules from primary hemostasis (Wurtzel et al., 2025). Together, these results emphasize that the engineering value is constrained amplification, and gating variables (background leakage, activation thresholds, DR, and shutdown or clearance) should be carried forward as core design objects for the gating section.

Target selection sets the safety boundaries. Low-specificity integrin-binding motifs such as Arg-Gly-Asp (RGD) often show weaker *in vivo* hemostatic efficacy than fibrin- or activated-platelet-targeting strategies, likely because nonspecific binding disperses particles to non-injured tissues and may interfere with endogenous GPIIb/IIIa function (Shuai et al., 2025). Thus, modular “strength” should be subordinated to lesion specificity and to how tightly activity can be constrained within the safety layer.

Translational deployability: from effective materials to clinical solutions. Deployability determines whether an effective material can become a clinical solution. For SynthoPlate, long-term stability after lyophilization and deployable administration routes such as intraosseous delivery support a shift from materials toward emergency-medicine use, and the lack of obvious toxicity or systemic platelet dysfunction across a relatively wide dose range further strengthens translational feasibility (Srinivasan et al., 2024; Liu Z. et al., 2025) (Figure 5E). At the functional level, adhesion-aggregation drives localized construction, mechanical modules reinforce and remodel clots, and procoagulant amplification is reserved for strictly gated contexts. The objective is therefore restricted and verifiable reconstruction rather than maximal platelet likeness, judged by potency, systemic-safety, and termination metrics that decouple hemostatic benefit from systemic thrombotic or immunological risk.

## 5 Gating design: triggers, thresholds, and deactivation

**FIGURE 6:**
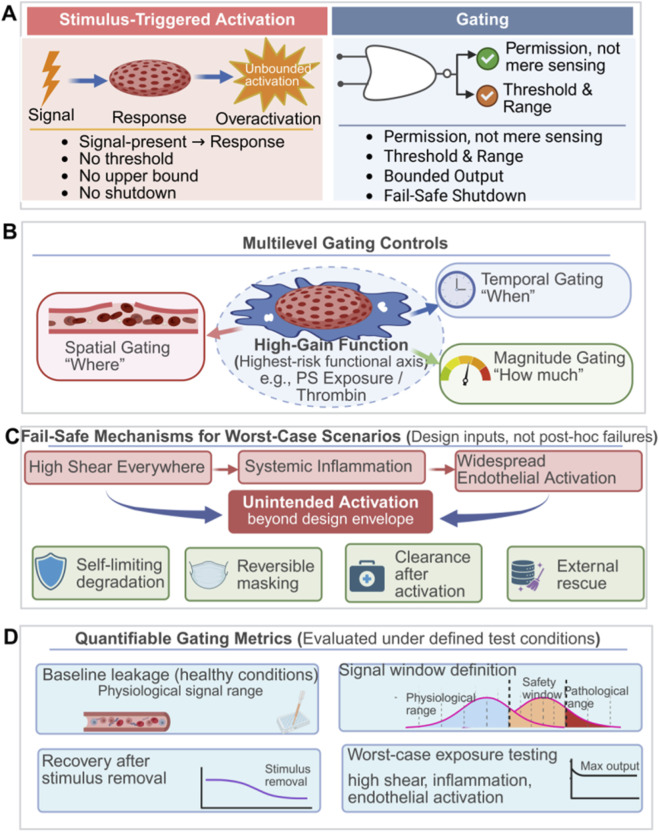
Engineering gating strategies for safe and translatable platelet-inspired therapeutic systems. **(A)** Stimulus-triggered activation versus permission-based gating. **(B)** Multilevel gating controls of high-gain, high-risk platelet-inspired functions. **(C)** Fail-safe mechanisms under worst-case pathological scenarios. **(D)** Quantifiable metrics for evaluating and validating gating performance. (Created with www.biorender.com).

Gating has shifted from an auxiliary tool for local efficacy to a core module that enables safe translation of platelet-mimicking nanotherapeutics. Because platelet-relevant hemostasis and immunomodulation are spatiotemporally constrained, systems must be hyporesponsive in circulation yet rapidly activate at lesions. Thus, the key problem is controlling where and when functions occur, with worst-case behavior kept within verifiable bounds. A robust design separates localization from activation: targeting enriches at lesions, while stimulus-responsive elements authorize functional output only in defined pathological contexts. In this way, gating binds high-reactivity exposure to a permission window and decouples efficacy from systemic thrombotic risk (Chee et al., 2024; Li et al., 2024d). “Triggering” refers to event-driven activation once a signal appears, whereas “gating” treats pathological signals as permission conditions that bound where, when, and how strongly functions operate and incorporates self-limiting fail-safes for loss-of-control scenarios (Yang L. et al., 2023) (Figure 6A). In engineering terms, triggering is an on instruction, while gating is permissioned control with bounded gain and guaranteed shutdown. Table 2 therefore organizes schemes by permission signal, gating target, worst-case scenario, and shutdown strategy, and links each to validation models and release criteria to make designs falsifiable and releasable.

**TABLE 2:** Engineering control matrix for gated biomimetic platelet-based nanotherapeutic systems.

Gating archetype	Permissive signal	Gated functional axis	Risk tier	Worst-case scenario	Fail-safe/Deactivation	Validation focus	Ref.
Masking-unmasking of procoagulant surface	Fibrinolysis	PS-driven coagulation	Tier 3 (highest)	Systemic thrombosis	Mandatory enzymatic unmasking; re-masking/neutralization; clearance/removal backstop	Background PS leakage in circulation; peak thrombin (worst-case); τ_shutdown/stop-window	Sekhon et al. (2022)
ROS-permissioned anticoagulant gating	ROS (thrombus-associated)	Antithrombotic effect	Tier 2	Off-target bleeding	ROS-threshold-dependent release; capped efficacy	Bleeding time; PT/aPTT; systemic coagulation perturbation ceiling	Zhao et al. (2021)
Stage-programmed thrombolysis-antioxidation	Ischemia-reperfusion	tPA/ROS control	Tier 2	Reperfusion mismatch	Stage-specific release	Phase-resolved efficacy-risk curves; ROS levels during reperfusion	Guo et al. (2022)
TME-permissioned toxicity containment	TME (pH/GSH)	ROS/metal toxicity	Tier 2	Systemic toxicity	Microenvironment-restricted	Leakage under normal tissues; maximal systemic exposure	Ren et al. (2024), Xin et al. (2025)
Dual endogenous-exogenous gating	Ultrasound + lesion signal	Effector release	Tier 2	Global mis-triggering	Reversible sequential gating	On/off ratio; activation-deactivation time constant	Li et al. (2025a), Zhao et al. (2025)
Behavior-programmed coagulation recruitment	Tumor coagulation niche	Local thrombosis	Tier 3 (high)	Runaway coagulation	Insufficient fail-safe (high risk)	Maximal exposure (worst-case); irreversibility check; τ_shutdown + containment required	Wang et al. (2023c)
Trigger-only self-amplifying assembly	Non-specific	Artificial coagulation	Tier 3 (failure)	Irreversible thrombosis	Absent (no fail-safe)	Uncontrolled coagulation under worst-case models; lack of shutdown	Yang et al. (2020b)
Low-risk cargo-release control	ROS/wound	Payload release	Tier 1	Leakage	Conditional release	Background leakage rate; therapeutic window	Chee et al. (2024), Zhang et al. (2025d)

Abbreviations: PS, phosphatidylserine; ROS, reactive oxygen species; PEG, polyethylene glycol; tPA, tissue plasminogen activator; TME, tumor microenvironment; τ_shutdown, characteristic shutdown time constant.

Gating stringency should be stratified by functional risk. High-gain procoagulant amplification, especially PS exposure, carries the greatest systemic-risk potential and thus requires stricter permission logic and explicit deactivation pathways than general release gating (Figure 6B). For example, fibrinolysin-gated PPNs pre-install PS but shield it in circulation with a cleavable polyethylene glycol (PEG) layer; only when fibrinolytic activity rises at injury sites is PS exposure permitted, enabling spatially constrained thrombin amplification with a translationally meaningful fail-safe (Sekhon et al., 2022). A similar logic applies to antithrombotic therapy, where elevated reactive oxygen species (ROS) in thrombus microenvironments can serve as an endogenous permission signal to release anticoagulant effects locally and reduce systemic bleeding risk (Zhao et al., 2021). Accordingly, along high-risk axes, stimuli responsiveness is a prerequisite for clinical acceptability rather than an optional optimization (Zhao et al., 2021; Sekhon et al., 2022).

When responsiveness expands from single triggers to multilevel amplification, it becomes behavior programming that reshapes system-level behavior by sculpting a responsive environment and allocating actions in time. Tumor-selective thrombosis strategies using truncated tissue factor (tTF)-RGD to initiate coagulation and build a recruitment field illustrate a shift from waiting for signals to constructing a signal landscape (Wang et al., 2023c). In tumor therapy, tumor microenvironment (TME) permissions such as acidic pH or high glutathione (GSH) are commonly used to bound toxicity, allowing platelet-membrane-wrapped systems to confine metal-ion release or ROS cascades to tumor conditions (Ren et al., 2024; Xin et al., 2025). Exogenous physical stimuli (e.g., ultrasound) offer strong temporal and intensity control, but selectivity depends on operational windows; thus, more translatable designs often overlay exogenous triggers with endogenous permissions to reduce false activation (Yang M. Y. et al., 2023; Zhao et al., 2025). Algorithm-assisted gating adds a computational layer but is limited by data quality, generalization, and workflow compatibility, and is better positioned to complement rather than replace pathology-adaptive gating (Zhang Y. et al., 2025).

Algorithm-assisted gating can be framed as a design-test-learn layer that supports early-stage prediction and tuning of permission windows before high-cost *in vivo* studies (Kulkarni et al., 2025; Lam et al., 2025). By coupling simplified hemodynamic and microenvironment models with ML surrogate models, formulation variables such as ligand density, shielding thickness, and cleavage kinetics can be mapped to gating outcomes across heterogeneous shear, oxidants, pH, and enzymatic activity (Barzegar Gerdroodbary and Salavatidezfouli, 2025; Kulkarni et al., 2025). This supports risk-aware threshold setting by estimating lesion-level activation versus off-target activation, and enables multi-objective optimization to maximize efficacy while constraining systemic liability (Kulkarni et al., 2025). The same computational layer can quantify key control metrics emphasized here, including background leakage, trigger threshold and DR, and a deactivation or shutdown time constant, and can propagate uncertainty to yield conservative bounds (Samei, 2025). Worst-case stress testing can be extended to digital stress testing by scanning scenarios such as high shear, systemic inflammation, and widespread endothelial activation *in silico*. This helps prioritize designs with bounded exposure and reliable shutdown (Pammi et al., 2025; Samei, 2025).

In stage-wise pathologies, spatial gating alone can create functional mismatch, making temporal gating a higher-order objective. A thrombus-specific platform that releases tissue plasminogen activator (tPA) via pH triggering to recanalize during ischemia and then releases antioxidants during reperfusion to scavenge ROS exemplifies stage-allocated function release (Guo et al., 2022). More generally, gating must specify what to release when to balance efficacy and risk (Guo et al., 2022; Zhang et al., 2025b). An anti-pattern is activation without reliable shutdown: CD105-targeted pNPs convert morphology and self-amplify to induce artificial coagulation, but lack reversible, lesion-specific permission conditions and fail-safe constraints that bound procoagulant amplification once triggered (Yang P. P. et al., 2020). The tension between stacking multiple stimuli and maintaining clear logic is a translational bottleneck (Yang L. et al., 2023), motivating explicit worst-case scenarios and fail-safe mechanisms as design inputs (Figure 6C).

Overall, gating design is not about triggering more, but about risk-stratified, verifiable control.

For high-risk modules, designs should implement permission-based, self-limiting fail-safes for prothrombotic functions and define explicit thresholds with a temporal division of labor in complex pathologies. In addition, they should pair exogenous triggers with endogenous permissions to reduce false activation and front-load worst-case scenarios as the primary validation thread (Guo et al., 2022; Sekhon et al., 2022; Yang L. et al., 2023; Yang M. Y. et al., 2023; Zhang et al., 2025b; Zhang Y. et al., 2025; Zhao et al., 2025). Performance should be quantified by background leakage, trigger threshold and DR, and a deactivation or shutdown time constant. Validation should be anchored in worst-case stress testing that measures maximal effector exposure and bounds systemic coagulation and immune perturbation under high shear, systemic inflammation, or widespread endothelial activation, making safety boundaries falsifiable and releasable. Key metrics are summarized in Figure 6D.

Worst-case stress testing can be operationalized by anchoring validation to three canonical contexts highlighted by the reviewer. In high-shear arterial environments (e.g., stenosis-mimicking flow with vWF-rich substrates), the key risk is unintended propagation from capture to aggregation. Evaluation should therefore emphasize shear-capture thresholds, adhesion kinetics, and integrin-activation surrogates. These readouts can be interpreted as a trigger threshold plus dynamic range that remains quiescent under baseline circulation (Watson et al., 2025; Zeibi Shirejini et al., 2025). In systemic inflammatory states (complement/NET-prone or cytokine-primed milieus), the dominant risk is background immune-thrombotic drift; therefore, baseline procoagulant leakage and thrombin-generation ceilings are appropriate boundary readouts, framed as bounded Δ-perturbations versus matched controls (Josefsson et al., 2023; De Nardi et al., 2024; Zeibi Shirejini et al., 2025). Under widespread endothelial activation (inflamed endothelium mimics with elevated adhesion ligands), the risk shifts to diffuse off-target adhesion and consumptive sequestration. In this setting, off-target adhesion indices and persistence/clearance proxies should be paired with explicit termination conditions (stop-windows). These stop-windows enforce return to a low-effector state once permissive cues dissipate (Dupuy et al., 2025; Qiu et al., 2025). Across the worst-case contexts defined above, Tier-3 (highest-risk) designs must additionally satisfy an explicit shutdown requirement to prevent persistence of effector exposure beyond the permissive window. Operationally, we specify shutdown at two levels. Functional shutdown requires that high-gain effector axes (e.g., PS-driven procoagulant presentation or artificial amplification) are default-off in circulation, become permissive only within a bounded window, and return to a low-effector baseline within a defined stop-window (τ-shutdown) once permissive cues dissipate (Stalker, 2024). System-level containment provides an independent safeguard under loss-of-control scenarios by limiting exposure via actionable containment routes (such as accelerated clearance, sequestration, capture, or an externally enabled reversal channel), thereby preventing prolonged systemic exposure even if functional deactivation is delayed (Bussin et al., 2025). In validation, Tier-3 pass/fail should therefore include maximal-exposure ceilings under the worst-case panels defined above (e.g., PS/leakage and thrombin-generation ceilings) together with a demonstrated τ_shutdown/stop-window, and an irreversibility check when self-amplifying elements are present (Josefsson et al., 2023; Haisma et al., 2024).

## 6 Disease-oriented applications: roles, risk gradients, and translational boundaries

Platelet-mimicking nanotherapeutics span hemorrhage, vascular inflammation and ischemia-reperfusion, cancer, infection, and drug reversal. Translation across indications hinges on permissioned activation, bounded spillover, and releasable CQAs/CMC criteria that balance efficacy and safety. Using the structure-membrane-function-gating framework, we map applications along a risk gradient: high-gain execution (hemostasis/reversal), navigation-centered modulation (vascular inflammation/ischemia-reperfusion (I/R), and multilayer-gated de-risking to limit positive feedback (cancer/infection). Table 3 summarizes roles, risk drivers, and translational boundaries.

**TABLE 3:** Engineering roles and translational boundaries of biomimetic platelet nanotherapeutics.

Application context	Engineering role (risk gradient)	Biomimetic layers (S/M/F/G)	Core engineering strategy	High-gain modules/Failure risks	Gating and de-risking design	Efficacy endpoints	Translational boundaries	Assay panel (surrogates)	Acceptance window form	Ref.
Acute hemorrhage	Executor (high gain)	S√ F√ G△	Platelet-mimicking procoagulant nanoparticles to accelerate clot formation	Systemic thrombotic amplification	Injury-/shear-associated activation	Hemostasis time, blood loss	Narrow dose window; reversibility required	MF hemostasis; clot strength; TGA	TGA ceiling; AV ceiling; Δ-bound PA	Sekhon et al. (2022)
Acute hemorrhage	Executor (high gain)	M√ F√ G△	Platelet-activated nanoparticles for thrombin delivery	Thrombin spillover	Vascular injury targeting	Hemostatic efficiency	Coagulation cascade amplification	Injury-targeting adhesion; thrombin activity/retention; TGA	Thrombin spillover ceiling; TGA ceiling; dose-time cap	Girish et al. (2020)
Thrombolysis	Navigator (constrained execution)	M√ F√ G√	Platelet membrane-coated nanotubes for accelerated thrombolysis	Hemorrhagic complications	Thrombus targeting + spatial confinement	Recanalization rate	Systemic bleeding risk	Thrombus-targeting MF; fibrinolysis potency; TGA	Thrombus-only threshold + DR; off-target lysis ceiling; Δ-bound hemostasis	Liu et al. (2023a)
Thrombolysis	Navigator	M√ F√ G√	Functionalized platelet membrane NPs to reduce bleeding risk	Off-target fibrinolysis	Selective thrombus release	Thrombolysis/bleeding ratio	Targeting stability	Lesion binding/retention; MF thrombus confinement; PK/BD stability	Targeting stability (comparability) window; bleeding ceiling; Δ-bound PA	Wang et al. (2020b)
Thrombolysis	Navigator	M√ F√ G√	H_2_O_2_-responsive platelet membrane-coated NPs	ROS mis-triggering	Lesion-specific ROS gating	Thrombolytic efficacy	Specificity under inflammation	Trigger-response curve; MF thrombus gating; TGA	Trigger threshold + DR; mis-trigger ceiling; Δ-bound coagulation	Zhao et al. (2021)
Antithrombotic reversal	Executor (constrained)	S√ M√ F√ G√	Engineered nanoplatelets for tPA delivery	Dose-dependent hemorrhagic risk	Dose and temporal capping	Thrombus reversal	Deployment and stop-window	Reversal potency; onset/termination kinetics; TGA	Dose-time cap; stop-window (τ-shutdown); bleeding ceiling	Xu et al. (2020)
Traumatic bleeding	Executor	S√ F√ G△	Lyophilized synthetic platelets for intraosseous infusion	Prothrombotic risk	Spatial restriction (bone marrow)	Survival rate	Manufacturing and storage advantages	MF primary hemostasis; PK/BD; PA markers	Spatial confinement window; Δ-bound PA; circulation-time ceiling	Liu et al. (2025c)
Atherosclerosis	Navigator	M√	Platelet membrane-coated Fe_3_O_4_ nanoparticles for vulnerable plaque recognition	Platelet activation	Diagnostic/imaging-oriented use	Plaque localization	Long-term immune and coagulation safety	Plaque-binding potency; imaging S/B; PA markers	Δ-bound PA; repeat-dose immunogenicity window; organ-burden ceiling	Li et al. (2024c)
Ischemic stroke	Navigator	M√ F△ G√	Modular biomimetic nanobubbles regulating thrombo-inflammation	Microthrombosis	Cascaded gating	Infarct volume reduction	Systemic inflammation background	Thrombo-inflammation markers; MF microthrombosis surrogate; Comp panel	Cascaded trigger thresholds; microthrombosis ceiling; Δ-bound Comp/coagulation	Li et al. (2025a)
Ischemic stroke	Navigator	M√ F△ G√	Magnetic platelet membrane NPs for targeted NO delivery	NO spillover	Magnetic targeting + *in situ* generation	Neurological recovery	External field controllability	Targeting under field; NO release/flux; PA/TGA	External-control window; NO spillover ceiling; Δ-bound PA/coagulation	Li et al. (2020b)
Cancer	Buffer-gated	M√ F△ G√	Modulating acidosis/hypoxia to sensitize chemotherapy	Off-target delivery	TME-permissive release	Tumor growth inhibition	Repeated dosing safety	TME trigger-response; BD/tumor enrichment; repeat-dose safety panel	TME-permissive threshold + DR; off-target exposure ceiling; repeat-dose coag/immune Δ-bound	Luo et al. (2022)
Cancer	Buffer-gated	M√ F√ G√	Co-delivery of anti-PD-1 and sorafenib	Immunothrombosis	Multilayer gating	Tumor suppression	Immune-related toxicity	Immuno-tox panel; coag/PA panel; BD	Immunothrombosis ceiling; immune-tox ceiling; Δ-bound coagulation	Da et al. (2024)
Cancer	Buffer-gated	S√ F√ G√	Mitochondrial respiration inhibition to induce ICD	Systemic toxicity	Dose capping	Immune activation	Metabolic safety	ICD surrogates; systemic tox panel; coag/PA	Dose cap; systemic-tox ceiling; Δ-bound PA/TGA	Mai et al. (2020)
Cancer	Buffer-gated	M√ F√ G√	Dual GSH depletion to enhance radio-immunotherapy	ROS amplification	TME-specific gating	Synergistic efficacy	Redox homeostasis	ROS trigger-response; oxidative-stress biomarkers; coag/Comp	TME-specific threshold + DR; ROS amplification ceiling; redox-homeostasis Δ-bound	Li et al. (2024b)
Systemic infection	Buffer-gated	M√	Platelet membrane NPs blocking *S. aureus* virulence	Coagulation-inflammation loop	Decoy-based de-risking	Survival benefit	DIC risk	Pathogen neutralization potency; DIC biomarkers; Comp panel	Coag-inflammation loop ceiling; Δ-bound coagulation; organ-injury ceiling	Kim et al. (2021)
Wound infection	Buffer-gated	M√ F△	Endotoxin adsorption + antibacterial action	Local inflammation	Adsorptive dominance	Wound healing	Localized application	Endotoxin adsorption capacity; local inflammation score; local coag markers	Localized-application window; systemic exposure ceiling; Δ-bound coagulation	Peng et al. (2021)
Pulmonary infection	Buffer-gated	M√	Activated platelet membrane vesicles for antibacterial therapy	Platelet activation	Dose regulation	Bacterial clearance	Lung distribution	Lung deposition/retention; bacterial clearance; PA markers	Comparability window; lung-distribution window; platelet-activation Δ-bound; dose cap	Wei et al. (2025)
Infection	Buffer-gated	M√	Hybrid cell membrane nanocarriers with triple-action strategy	Unpredictable immune-coagulation crosstalk	Multimembrane balance	Clearance efficiency	Batch consistency and regulatory complexity	Membrane-composition comparability; Comp/coag panel; PK/clearance	Comparability window; immune-coag crosstalk Δ-bound; clearance τ	Zhang et al. (2025a)
Lung injury	Buffer-gated	M√	Inhalable platelet vesicle-decoy nanoparticles	Coagulation suppression	Local delivery	Inflammation score	Inhalation safety	Aerosol performance; lung-local anti-inflammatory potency; systemic coag panel	Inhalation safety window; systemic coag suppression ceiling; lung retention ceiling	Jin et al. (2022)
Acute lung injury	Buffer-gated	M√	Inhibition of platelet activation and NET formation	Immunothrombosis	Suppressive biomimicry	Lung injury index	Long-term immune effects	NETs markers; inflammation index; coag/Comp panel	Immunothrombosis ceiling; Δ-bound NETs/coag; repeat-dose immune window	Li et al. (2025b)
Drug reversal	Executor (safety-prioritized)	M√ F√ G√	Platelet-mimicking nanosponges for antiplatelet drug reversal	Thrombosis	Adsorption without activation	Bleeding correction	Reversal controllability	Adsorption capacity; occupancy recovery kinetics; PA/TGA	Capacity-limit spec (ceiling); Δ-bound PA; over-reversal ceiling	Xu et al. (2023)
Drug reversal	Executor (safety-prioritized)	M√ F√ G√	P2Y12-overexpressing membrane NPs for ticagrelor reversal	Over-neutralization of antiplatelet activity	Competitive binding	Functional recovery	Engineered membrane regulation	Competitive binding potency; functional recovery assay; PA/TGA	Comparability window; over-neutralization ceiling; onset/offset window (τ); Δ-bound PA	Guo et al. (2024)
Traumatic hemostasis	Executor (material-level)	S√ F√ G√	Supramolecular material releasing platelet substitutes	Local thrombosis	Humidity-triggered, detachable release	Hemostasis time	Defined deployment, reversibility, and scalability	Trigger-response; local deposition/retention; MF hemostasis	Trigger threshold + DR; detachment τ; local thrombosis ceiling	Zeng et al. (2025)

Numeric acceptance windows are indication- and tier-dependent and should be finalized during process validation and comparability assessment.

Abbreviations: S, structural biomimicry; M, membrane biomimicry; F, functional biomimicry; G, gating strategies; NP, nanoparticle; UTMD, ultrasound-targeted microbubble destruction; NO, nitric oxide; DIC, disseminated intravascular coagulation; MF, microfluidics; TGA, thrombin generation assay; AV, annexin V (PS, exposure); PA, platelet activation markers (CD62P, PAC-1); Comp, complement (C3a/C5a/SC5b-9); PK/BD, pharmacokinetics/biodistribution; τ, time constant; GSH, glutathione; P2Y12, purinergic receptor P2Y12; Executor, high-gain execution role; Navigator, targeting and spatial navigation role; Buffer-gated, safety-prioritized buffering and conditional activation role.

Symbols: √ indicates the presence of a biomimetic layer; △ indicates a constrained or partial implementation.

### 6.1 Hemostasis and thrombus regulation: efficacy-risk-translation

Within the “multi-level biomimicry-gated control” framework, hemostasis and thrombus regulation are highly risk-sensitive (Raghunathan et al., 2022). Hemostasis requires injury-confined threshold crossing, whereas antithrombotic/thrombolytic effects must be thrombus-confined without systemic bleeding. Translational viability hinges on spatial permission, shear/thrombin/matrix gating, and dose-time windows. These are implemented across the structure-membrane-function-gating space (near-wall access, routing, execution, and spillover capping with deactivation/reversal).

For hemostasis, the engineering core is “threshold-like amplification plus localized execution.” In anticoagulant-associated bleeding, DOAC-related initiation/propagation bottlenecks can leave residual endogenous thrombin potential insufficient for rapid platelet-mediated amplification. Factor supplementation may restore endogenous thrombin potential, but it can also increase systemic coagulability and thrombotic risk (Shaw et al., 2023). Thus, the target shifts from “how much thrombin” to “whether amplification occurs at the right place and time.” A small subset of procoagulant platelets provides PS-mediated assembly interfaces to drive thrombin bursts and stabilize plugs; loss of spatial/temporal constraints can slide the system toward microthrombosis and thromboinflammation (Reininger et al., 2006; Denorme and Campbell, 2022). High arterial shear further accentuates the “high-gain yet must-be-constrained” nature: vWF-GPIb capture can gate entry and amplify coagulation in micro-injury zones, but may seed thrombosis if activated off-target (Reininger et al., 2006). Stage-wise regulation likewise indicates high-shear phases dominate initiation, followed by stable adhesion/aggregation shaped by deceleration, matrix exposure, and microenvironmental changes (Ruggeri and Mendolicchio, 2007). Accordingly, design should enforce injury-confined threshold crossing using shear/coagulation signals as permissions and impose dose-time ceilings to prevent spillover, avoiding broad adhesion and sustained procoagulant drive (Reininger et al., 2006; Ruggeri and Mendolicchio, 2007; Denorme and Campbell, 2022; Shaw et al., 2023).

At the execution layer, strategies largely fall into network-internal execution versus synergistic amplification. Network-internal approaches anchor function within fibrin networks and leverage mechanical remodeling for retraction-like effects. Ultra-soft PLPs based on ultralow-crosslinking poly (N-isopropylacrylamide) microgels enrich via fibrin-binding ligands, reorganize fibrin networks, and show no obvious systemic prothrombotic risk in rodent and large-animal studies (Nellenbach et al., 2024). Consistent with a “Brownian-wrench” mechanism, clot stiffness and densification can be increased without enhancing thrombin generation, and can be accelerated by local low-dose ultrasound (Nandi et al., 2019; Nandi et al., 2020a). By contrast, synergistic amplification accelerates early hemostasis by boosting endogenous platelet adhesion/recruitment (e.g., representative multi-ligand PLGA systems such as PLGA-PEG multi-ligand nanoparticles, PLGA-PSP) (Fu et al., 2025), but greater biomimetic complexity (discoid deformable, multivalent systems) can trade speed for local microthrombus risk, underscoring that targeting alone is insufficient for safety (Anselmo et al., 2014). Conditional activation helps decouple efficacy from off-target retention: TS-PLPs remain circulation-inert via thrombin-sensitive crosslinks and switch morphology/mechanics at injury sites to restore fibrin binding and retraction (Chee et al., 2024), while modular extensions such as Ag-PLPs add antibacterial activity without new procoagulant/thrombotic abnormalities (Chee et al., 2020). Overall, network-internal execution prioritizes safety via spatial constraint, whereas synergistic amplification prioritizes speed but relies more on gating logic, dose windows, and verifiable deactivation/reversal.

Beyond execution-layer substitution or amplification, some systems intervene deeper in coagulation cascades (fibrin assembly, thrombin generation, and platelet-activation signaling). Because efficacy and risk often co-amplify at cascade nodes, pro-/anti-coagulant activity should be injury-coupled and locally prioritized through spatial, temporal, and dose constraints. Intervention depth forms a spectrum with progressively narrower deployable windows, structural-assembly interventions are broader (e.g., knob B-mimetic microgels accelerating fibrin assembly) (Nandi et al., 2021). Signal-permission strategies are intermediate, exemplified by circulation-inert systems activated by platelet-derived injury signals (Srinivasan et al., 2024). Direct engagement of core amplification nodes are the narrowest and most dependent on gating plus deactivation (e.g., direct enhancement of thrombin generation and fibrin deposition) (Sekhon et al., 2022). Silk-fibroin microspheres similarly emphasiz anchoring amplification to recognizable injury interfaces (Shuai et al., 2025). In parallel, targeted routing uses membrane-mimetic interfaces to reduce systemic exposure (e.g., directing thrombin to injury regions) (Girish et al., 2022), while platelet-derived nanovesicles suggest “interface retained, contents stripped” may help decouple hemostasis from inflammation. However, these approaches raise membrane-source consistency and safety-control challenges (Jung et al., 2019; Girish et al., 2022). Overall, deeper intervention implies narrower windows and greater reliance on permission triggering and reversible deactivation to preserve local priority.

In contrast, antithrombotic and thrombolytic applications combine spatial routing with layered gating. Platelet-mimicking systems can localize anticoagulant or fibrinolytic activity to clots via adhesion or homing, but the same fibrin/activated-platelet recognition can increase off-target exposure and bleeding risk. Accordingly, translational viability thus hinges on thrombus-only permission, thrombus-relevant trigger or gating logic, and defined dose-time windows that are validated under worst-case conditions. Practical approaches include membrane-homing restriction, exogenous on-demand triggers, delivery-limited intrathrombus transport, and microenvironment-enabled local release (Wang et al., 2020b; Xu et al., 2020; Zhao et al., 2021; Li S. et al., 2022; Chen et al., 2023; Liu B. et al., 2023; Zhao Z. et al., 2023; Hua et al., 2024; Xiao et al., 2024).

In translational settings, evaluation shifts from stronger effects to deployable, measurable, and reversible risk boundaries. Biomimetic systems improve stability, supply-chain independence, and dosing flexibility, reconstructed execution can perturb blood homeostasis and must be governed by quantifiable risk controls (Wang et al., 2018; Girish et al., 2020; Nellenbach and Brown, 2022; Fernandez-Borbolla et al., 2024; Xu et al., 2024; Liu Z. et al., 2025). SynthoPlate illustrates this balance. Its lyophilized, peptide-functionalized lipid nanoparticles reconstruct primary hemostasis and enable room-temperature storage, rapid reconstitution, and intraosseous delivery, without obvious coagulopathy or organ toxicity across a relatively wide dose range (Liu Z. et al., 2025). However, high-gain adhesion/aggregation/procoagulant modules can turn prothrombotic under systemic dosing or dose deviation. Thus, translatable designs should be “enough yet constrained”: define indication-specific ceilings on circulation time and dose (especially for PS-like amplification interfaces) and incorporate gating or deactivation that anticipates worst-case scenarios (Wang et al., 2018; Raghunathan et al., 2022; Fernandez-Borbolla et al., 2024; Xu et al., 2024).

Accordingly, translational boundaries reduce to three checks: worst-case safety margins, reversibility via deactivation/clearance, and manufacturing consistency with defined CQAs and release criteria. Release priorities include minimal background procoagulant leakage, bounded coagulation perturbation, predictable biodistribution/clearance, batch-consistent composition and function, stable membrane-protein retention/orientation, and a clinically usable reversal channel as a second safety threshold (Wang et al., 2018; Girish et al., 2020; Xu et al., 2020; Nellenbach and Brown, 2022; Fernandez-Borbolla et al., 2024; Xu et al., 2024; Liu Z. et al., 2025).

### 6.2 Vascular disease and sterile inflammation: platelet-mimicking strategies

In vascular disease and sterile inflammation, platelet-vascular interactions are continuous, making platelet-mimicking systems most suitable as navigation/entry modules that localize lesion-specific, low-prothrombotic effector actions and enforce predefined safety boundaries (spillover, inflammatory amplification, organ burden). This maps onto structure-membrane-function-gating control over near-wall access/shear matching, interface recognition/routed entry, and gating that restricts aggregation/procoagulant and systemic immune activation to permitted windows (Fang et al., 2022).

Atherosclerosis and restenosis: navigation-effector decoupling under repeat-dosing boundaries. Prioritize vascular-interface targeting for local immunomodulation and repair over hemostatic execution: keep adhesion/recognition and immune entry for enrichment, but avoid aggregation and procoagulant interfaces to limit spillover. Under chronic repeat dosing, immune activation and hepatosplenic burden are primary safety limits. Platelet-membrane Fe_3_O_4_@PLGA demonstrates pathway-level interface matching for vulnerable-plaque imaging and localization (Li et al., 2024c). Building on navigation, plaque modulation can decouple localization from effector output. Platelet membrane-coated M2 macrophage-derived extracellular vesicles use platelet membranes for targeting and entry while leveraging the microRNA (miR)-99a-5p axis to suppress inflammation and foam-cell formation without coagulation abnormalities (Xie et al., 2024). Related navigation-enabled anti-oxidative and anti-inflammatory modules (polyphenol-CeO_2_ nanozymes; selenium/ginsenoside Rb1) improve metabolic dysregulation and plaque stability (Yin et al., 2022; Li et al., 2024d). Restenosis also favors a navigation-effector decoupling strategy aimed at vascular healing. For example, interleukin-10-gene-engineered platelet-mimicking nanoparticles increase injured-vessel targeting via glycoengineering while reducing liver and spleen clearance (Li F. et al., 2023). Functionally, these particles promote endothelial repair, suppress aberrant smooth-muscle proliferation, and induce macrophage M2 polarization (Li F. et al., 2023). In parallel, platelet-membrane-coated rapamycin nanoparticles stent coatings provide a complementary route to coordinate anti-proliferative control with vascular healing (Li Y. et al., 2023). Efficacy can be stage-dependent (e.g., limited benefit in late aortic-valve calcification with little reversal of established lesions), implying hard boundaries in indication selection and dosing windows (Geng et al., 2023). Overall, the core is not “more platelet-like,” but adhesion-based localization/entry plus systematic avoidance of aggregation/procoagulant interfaces. Repeat-dosing immunity, biodistribution/clearance, and prothrombotic spillover should be made falsifiable via QC and long-term follow-up. Stage dependence further constrains indications and windowing.

I/R: functional decoupling + microenvironment-permitted release. In I/R, designs should leverage adhesion or homing for lesion entry and early post-reperfusion control of inflammation and microcirculatory imbalance, while avoiding aggregation and cascade amplification. Platelet interactions with endothelium and immune cells drive inflammatory amplification, microthrombi, and microvascular dysfunction. Early after reperfusion, vWF/collagen exposure recruits platelets via GPIbα-vWF and GPVI-collagen and shapes no-reflow, creating a highly context-dependent spatiotemporal window (Wang L. et al., 2023). Accordingly, platelet biomimicry should prioritize entry without procoagulation and configure effectors as anti-inflammatory, anti-oxidative, or reparative outputs. Platelet membranes can serve as recognition interfaces for I/R-stressed endothelium. For example, a platelet-membrane-human plasma EV hybrid targets I/R-injured vasculature, increases microvascular density, suppresses apoptosis, and attenuates ventricular remodeling (Jiang J. et al., 2023). As a minimal navigation reference, biomimetic microbubbles enable sensitive molecular ultrasound imaging of early I/R injury without procoagulant or therapeutic functions (Bai et al., 2024). To bypass perfusion heterogeneity via immune entry, platelet-like fusogenic liposomes bind lymphocyte antigen six complex, locus C-positive inflammatory monocytes and traffic to injured myocardium (Tan et al., 2021). These liposomes deliver miR-21 to promote macrophage M2 polarization and improve long-term function. Similarly, platelet membranes carrying miR-181a-5p promote microvascular angiogenesis by suppressing c-FOS without procoagulant responses (Liu D. et al., 2025). For oxidative-stress and fibrosis pathways, microenvironment-permitted release can compress systemic exposure. Membrane-camouflaged PLGA nanoparticles delivering a miR-155-5p inhibitor restore nuclear factor erythroid 2-related factor 2 signaling (Wang et al., 2022), and GSH-triggered local H_2_S release suppresses oxidative stress and fibrosis without detectable aggregation or coagulation abnormalities (Chen et al., 2025b). Higher-complexity platelet-inspired “nano-cells” may improve cardiac function but complicate manufacturing consistency and long-term safety evaluation (Su et al., 2019). Overall, I/R designs should implement functional decoupling (entry and immunomodulation without copying procoagulant amplification). Key translational sensitivities include avoiding aggregation/procoagulant spillover under no-reflow and establishing reproducible evidence for manufacturing consistency, long-term distribution, and safety follow-up (Su et al., 2019; Wang et al., 2022; Jiang J. et al., 2023; Bai et al., 2024; Chen et al., 2025b; Liu D. et al., 2025; Shi et al., 2025).

Neurovascular pathology (AIS): precise localization + de-activation gating under blood-brain barrier (BBB) boundaries. Since AIS links platelet activation, thrombosis, inflammation, and BBB disruption, platelet biomimicry should prioritize intraluminal localization and de-activation gating rather than procoagulant enhancement, with explicit safety limits on BBB integrity/hemorrhagic transformation, systemic coagulation, microthrombus burden, and hemodynamics. Annexin V platelet-membrane mimetics targeting PS can inhibit further platelet activation and, when carrying tPA, improve perfusion and reduce infarct volume while lowering BBB permeability versus free tPA (Quan et al., 2022). Effector configurations should center on microcirculation and BBB risk, including immunomodulatory nanobubbles (Li M. et al., 2025), NO-generating magnetic nanocarriers that restore local flow under external fields with reduced systemic hypotension risk (Li M. et al., 2020), and lumbrokinase delivery that preserves thrombolysis at lower systemic coagulation interference (Wang et al., 2020b). Low-interference “hitchhiking” paths include T3 nanoparticles for neuroprotection and reduced BBB damage (Syeda et al., 2025)and deferoxamine delivery for ferroptosis inhibition in the penumbra without major coagulation perturbation (Wang et al., 2025). High-mobility group box 1-driven neutrophil extracellular traps (NETs) formation supports adjunct strategies that inhibit NETs or inflammatory cascades (Denorme et al., 2022). Overall, platelet-membrane mimetics appear more promising for intravascular targeting and neurovascular-unit protection than for deep brain parenchymal delivery in stroke (Liu Z. et al., 2023; Chen X. et al., 2025).

### 6.3 Tumor microenvironment: functional leverage and immune remodeling

Tumor-associated platelets both drive metastasis and immune suppression and provide engineerable adhesion and damage-responsive homing. Accordingly, platelet-mimicking tumor nanotherapeutics should be designed as navigation plus locally activated intervention under hard control constraints, with worst-case thrombosis/immunothrombosis and immune spillover translated into releasable safety criteria spanning coagulation/platelet perturbation, microthrombus burden, complement-inflammatory profiles, and biodistribution, clearance, and repeat-dosing immunogenicity (Li S. et al., 2024).

Tumor-associated platelets function as active regulatory nodes linking coagulation, inflammation, immunity, and tumor biology (Li et al., 2018). Tumor cells can trigger tumor cell-induced platelet aggregation and exploit platelet-mediated immune evasion (e.g., major histocompatibility complex class I transfer and transforming growth factor-β [TGF-β]-related suppression of NK/T cells), while platelet-derived mediators also reshape vascular permeability and metastatic niches (Zhao J. et al., 2023; Zhou et al., 2023; Li S. et al., 2024). Locally, platelet factors and vesicles reinforce immunosuppressive microenvironments through multiple axes, promoting Tregs, myeloid-derived suppressor cells, and pro-tumor macrophages (Zhou et al., 2023; Mittelheisser et al., 2025). Engineering leverage lies in adhesion-based recognition and residence windows, *in vivo* stability linked to immune-evasion signaling, and responsiveness to inflammation/damage cues. However, these same interfaces are coupled to pro-metastatic, immunosuppressive, and hypercoagulable tendencies. Permissioned gating and fail-safes are therefore required, especially under long-term or repeat dosing when “education” effects can further narrow the safety window (Li S. et al., 2024; Mittelheisser et al., 2025).

Navigation (arrival): transferable delivery shells with bounded risk. In tumor-targeted nanotherapy, the bottleneck is often achieving sufficient tumor exposure at the right time and place rather than potency alone. Platelet biomimicry repurposes adhesion, immune evasion, and damage-responsive recognition into reusable interfaces, improving stability and delivery compatibility without requiring high-density artificial ligands (Wan et al., 2022; Wu Y. et al., 2024). Platelet membranes preserve adhesion axes (e.g., P-selectin/CD44-related interactions) and CD47-mediated anti-phagocytosis, enabling tumor recognition and prolonged circulation that enhance enrichment and delivery efficiency (Wu Y. et al., 2024). These gains appear transferable across CD44-high tumors, with cross-tumor and cross-payload validations supporting partial generalizability as a “universal delivery shell” (Wan et al., 2022; Jiang H. et al., 2023).

Residence and local activation: from enrichment to controllable efficacy translation. Because tumors are often procoagulant and immunothrombotic, enrichment alone may not translate into efficacy, while aggregation/procoagulation mimicry can increase thrombosis risk. Thus, retain navigation but blunt hazardous axes, and gate tumor-local activation with deactivation-defined safety limits. Residence can be extended by exploiting damage-induced collagen exposure and inflamed endothelium as re-recruitment cues for cascade enrichment and vascular “locking,” compatible with anti-angiogenic strategies (Li et al., 2021; Ding et al., 2023). Tumor-local triggering can be added via acidity- or redox-responsive release while preserving circulation and adhesion advantages (Wan et al., 2023).

Intervention as threshold regulation: controllable synergy over reactive stacking. Microenvironment reprogramming that lowers response thresholds can convert platelet-mimetic platforms from delivery carriers into threshold-regulation units, for example by co-targeting acidity and hypoxia (Luo et al., 2022)or combining GSH depletion with sonodynamic activation to sustain intratumoral ROS with acceptable systemic safety (Huang C. et al., 2021). As reactivity increases, controllability becomes the central design requirement. Platelet-mimetic membranes can confine multimodal reactions (e.g., mild photothermal plus chemo or gas therapy) and localize oxygen/ROS generation to tumors (Ren et al., 2024; Wu D. et al., 2024). In parallel, membrane camouflage improves blood compatibility and buffers toxicity in highly reactive materials, including black phosphorus quantum dots (Shang et al., 2019; Liu et al., 2024). Catalytic platforms such as single-atom nanozymes can further lower tolerance thresholds by suppressing heat-shock responses via mitochondrial damage, linking energy, reaction, and biological response (Qi et al., 2022). Overall, the engineering sequence can be summarized in three stages. First is arrival (adhesion and immune evasion) (Wan et al., 2022; Jiang H. et al., 2023; Wu Y. et al., 2024), Second is residence, driven by damage or inflammation damage- or inflammation-mediated (Li et al., 2021; Ding et al., 2023). Third is intervention, defined by bounded and controllable threshold reprogramming (Shang et al., 2019; Huang C. et al., 2021; Luo et al., 2022; Qi et al., 2022; Liu et al., 2024; Wu D. et al., 2024). Translation remains limited by mouse-model dominance and demands stronger evidence for manufacturability and batch consistency, repeat-dosing immune and coagulation safety boundaries, and performance under clinical-like dosing (Huang C. et al., 2021; Liu et al., 2024; Wu D. et al., 2024).

Immune-event programming: spatial, temporal, and pathway control. Platelet-mimicking systems can help immunotherapy evolve from payload delivery to spatial, temporal, and pathway-level control of immune cascades by coupling TME pre-conditioning with damage and inflammation responsive adhesion interfaces that sustain amplification (Chen et al., 2020; Lv and Ma, 2025; Zhang Y. et al., 2025). For example, low-dose radiotherapy can prime the microenvironment to increase uptake of platelet-membrane mimetics and remodel vascular and matrix features, strengthening downstream photothermal or photodynamic effects (Chen et al., 2020). Under conditions of long circulation, anti-phagocytosis, and tumor adhesion, photodynamic therapy-induced immunogenic cell death (ICD) can synergize with programmed death-ligand 1 downregulation and hypoxia relief to drive dendritic cell maturation, CD8^+^ infiltration, and immune memory formation (Zhang Y. et al., 2025), consistent with broader reviews of platelet membranes or derived structures and engineered platelets or vesicles (Lv and Ma, 2025).

Temporal programming can be achieved by platelet-membrane-camouflaged co-delivery of anti-PD-1 and targeted drugs to reshape intratumoral immune composition and inhibitory programs (Da et al., 2024). Related ICI-independent designs pair photodynamic therapy-induced ICD with hypoxia relief (e.g., via mitochondrial respiration inhibition) to boost antigen presentation while limiting myeloid-derived suppressor cell and Treg recruitment (Mai et al., 2020). TLR7/8 agonists can further translate local activation into systemic immune memory with lower systemic toxicity (Bahmani et al., 2021). Pathway programming can also tune stress and death pathways. Examples include dual GSH depletion to amplify radiotherapy-driven ROS/ICD and trigger abscopal effects (Li X. et al., 2024), hypoxia-targeted delivery to reduce cancer stem-cell tolerance and suppress recurrence (Ning et al., 2023; Jiang et al., 2024a), and regulated cell-death (ferroptosis/cuproptosis) to enhance antigen presentation and immune activation (Jiang et al., 2020; Xin et al., 2025). These approaches can be supported by ROS-responsive schemes (Yan et al., 2022) and broader tumor recognition modules such as variant surface antigen 2-chondroitin sulfate A (Zhou et al., 2021).

High-gain paradigms require provable control. For example, strategies that induce selective thrombosis in tumor neovasculature to create immune-amplification fields and recruit anti-PD-1-conjugated platelets must show spatial confinement, temporal reversibility, and no widening of bleeding windows or increases in systemic thrombosis or immunothrombosis risk (Wang et al., 2023c). Accordingly, platelet biomimicry in tumor immunotherapy is most valuable for programming immune events in space, time, and amplification pathways, and translation should be judged by falsifiable, releasable limits on off-target thrombosis, inflammatory spillover, repeat-dosing immune responses, and biodistribution and clearance.

### 6.4 Infection and sepsis: opportunities and boundaries

Infection and sepsis couple immunity and coagulation nonlinearly: platelet-mimicking platforms can lower pathogen/toxin burden but may also feed platelet-NETs-coagulation loops that escalate to immunothrombosis and organ injury. Across the gain-risk gradient from decoy neutralization to high-gain programming, translatable designs require permissioned triggers, capped effects, and reversible deactivation, with safety bounded by platelet activation and platelet nanoclusters, NET burden, coagulation-fibrinolysis balance, complement/inflammatory signals, and organ distribution/perfusion.

Platelets show immunological duality in infection: they can support host defense in short, focal, reversible windows but become amplifiers of immunothrombosis under sustained inflammatory drive. They sense pathogens via pattern-recognition receptors and granule mediators (Semple et al., 2011), bind or encapsulate microbes and contribute antiviral effects (Li C. et al., 2020), and couple to innate immune cells through thrombotic and immune receptors (e.g., GPIIb/IIIa, GPIb, FcγRIIa, TLRs,) to promote recruitment, NET formation, and pathogen clearance (Li C. et al., 2020; Cox, 2023). In sepsis, persistent activation with PNC formation signals a tipping point where NET release, microvascular platelet aggregation, and endothelial injury self-reinforce, impairing perfusion; platelet count or function tracks severity nonlinearly, and sustained or severe thrombocytopenia predicts worse outcomes (Assinger et al., 2019). In the immunothrombosis framework, initially defensive intramicrovascular structures can escalate to widespread platelet activation, excessive NETs, coagulation amplification, disseminated intravascular coagulation (DIC), and organ injury (Engelmann and Massberg, 2013; Iba and Levy, 2018; Wienkamp et al., 2022). Engineering should therefore constrain engagement with permission triggers and verified termination or deactivation, benchmarked against baseline and worst-case immunothrombosis readouts.

Low-gain de-risking: decoy-like neutralization and buffering. Adsorbing pathogens or toxins provides a robust, low-risk entry point under coupled immunity and coagulation by reducing toxicity and inflammatory burden without intentionally amplifying immune cascades. Platelet-membrane PNPs exemplify this approach by sequestering pore-forming toxins (e.g., *S. aureus* α-toxin), thereby neutralizing circulating toxicity, protecting immune effectors, limiting excessive NET formation, and improving survival in methicillin-resistant *Staphylococcus aureus* (MRSA) systemic infection models with favorable safety (Kim et al., 2021). Moderate-gain strategies: adsorption plus locally bounded effectors. Augmenting adsorption with local antibacterial effectors increases efficacy but narrows safety margins. For example, CSO@PM preserves pathogen binding and LPS adsorption while adding photothermal and copper-based antibacterial activity to suppress inflammation and promote tissue repair in local infection models (Peng et al., 2021); systemic translation therefore requires stringent validation of dose windows, organ burden, and immunity-coagulation perturbations. Higher-gain amplification: NETosis and immune programming with narrow windows. Moving from adsorption to immune amplification sharply narrows safety margins because NETosis-linked gains can couple to immunothrombosis and tissue injury. Platelet volume illustrates this trade-off by combining toxin adsorption and bacterial binding with platelet-neutrophil amplification to induce NETs and improve clearance in pulmonary infection models, while also raising sepsis-relevant risks (Wei et al., 2025). Reviews therefore emphasize membrane-camouflaged buffering as a distinct de-risking route that adsorbs pathogen-associated molecular patterns, damage-associated molecular patterns, and inflammatory mediators to raise triggering threshold, with membrane source setting the risk floor (platelet and RBC relatively inert; immune-cell membranes more signaling-prone) (Wang X. et al., 2023; Wang et al., 2023d). Simplified designs such as platelet-derived nanodecoys prioritize decoy neutralization and improve survival in MRSA systemic infection without obvious platelet hyperactivation, NET amplification, or coagulopathy (Sun et al., 2024). Accordingly, a translatable path is de-risking first and amplification only with permissioned triggers, reversible termination or deactivation, and higher-order validation that immunothrombosis and organ-injury risks are not increased (Wei et al., 2025).

Local infection vs sepsis extrapolation: a hard boundary. In localized infection and biofilms, platelet-membrane navigation can localize antibacterial modules and enable microenvironment-triggered Ag-metal-organic framework or bimetallic metal-organic framework release and ROS synergy to improve exposure and tissue repair with limited systemic toxicity (Huang R. et al., 2021; Shi et al., 2024). Yet this “locally acceptable” amplification may fail in sepsis: if efficacy depends on strong catalysis, prolonged retention, or high-reactivity modules without strict spatiotemporal constraints, ROS amplification or metal-ion release can injure endothelium and platelets, amplify inflammation, and disrupt immunity-coagulation homeostasis (Shi et al., 2024).

Complex hybrid membrane systems and long-term retention further tighten boundaries. Stacking multiple membrane sources with antibacterial or regulatory modules can raise local efficacy, but mixed membrane immune attributes increase coupling and reduce predictability, allowing small preparation or inter-individual differences to expand into large biological uncertainty (Zhang et al., 2025a). Likewise, long-term retention or active motion can improve delivery in inhaled microrobots or lung-enrichment systems yet elevate inflammation or immunothrombosis risk in sensitive organs, so safety depends on tightly bounded dose, motion parameters, and time windows (Jin et al., 2022; Li Z. et al., 2025).

Finally, platelet biomimicry can serve as a risk reducer by competitive occupancy or signal buffering that suppresses platelet activation and NET formation, interrupts platelet-neutrophil feedback, and mitigates inflammation and tissue injury, supporting a sepsis-oriented principle of controllable de-amplification at key nodes rather than maximal immune activation or bactericidal gain (Li X. et al., 2025). Accordingly, amplification-oriented anti-infective designs should restrict amplification to local, controllable, reversible windows with built-in deactivation and must show bounded worst-case immunothrombosis and organ-injury risk as complexity and retention increase (Jin et al., 2022; Li Z. et al., 2025; Zhang et al., 2025a).

### 6.5 Reversal of antiplatelet/anticoagulant therapy: engineered control

With widespread antiplatelet and anticoagulant therapy, rapid and controllable reversal without eroding long-term antithrombotic benefit is a shared clinical and engineering challenge. Because inhibition is driven by systemic exposure and sustained receptor or drug occupancy, recovery after purinergic receptor P2Y12 inhibitors is often limited by platelet turnover, and reversal of DOACs or heparin-class agents is largely clearance-limited (Mega and Simon, 2015). Thus, downstream compensation can be unstable; under ticagrelor, free circulating drug can rapidly inactivate transfused platelets, decoupling transfusion volume from functional restoration (Bertling et al., 2018). Reversal should therefore be defined as bringing exposure and occupancy back below a hemostatic threshold within a set time window while preserving protection outside it.

Reversal should withdraw drug effects rather than enhance coagulation. Removing or competitively binding the drug lowers free levels and relieves receptor occupancy, while capacity limits and clearable or deactivatable designs cap intensity and ensure termination. Key risks include thrombosis from interface-triggered platelet activation, over-reversal, and nonspecific interactions without reliable shutdown (Cao et al., 2023). Accordingly, reversal designs require gate, cap, and fail-safe logic, with release evidence for clearance or binding kinetics and occupancy recovery, worst-case bounded onset and termination windows, and controlled biodistribution with minimal platelet or coagulation perturbation.

Among clinical approaches, small molecules such as ciraparantag can noncovalently complex multiple anticoagulant classes with minute-scale onset and short-term predictability in clinical studies (Ansell et al., 2022). However, they are closer to systemic neutralization, with capping and termination largely determined by dose and binding capacity. Biomimetic and nanoengineering approaches instead make reversal designable, cap-able, and terminable. Membrane-mimetic “drug sinks” use receptor repertoires as competitive adsorption interfaces, reducing reversal to “drug-receptor competition”; lacking intracellular signaling and secretion, they more readily decouple reversal efficacy from prothrombotic risk (Xu et al., 2023). Controllability and manufacturability can be further improved by tuning receptor composition, for example using membranes overexpressing P2Y12 receptors (Guo et al., 2024).

Supramolecular and porous-material routes pursue physicochemical neutralization or encapsulation, so capping follows directly from definable binding capacity. Heparin reversal can be reduced to charge-driven drug-polysaccharide neutralization (Weiss et al., 2015), while programmable receptors and porous polymers enable tunable recognition or “inclusion-isolation” with reduced nonspecific blood interactions (Huang et al., 2020; Lin et al., 2022). The major limitation is terminability: without deactivation or clearance mechanisms, over-reversal and nonspecific interactions become primary risks, so termination should be designed in on par with capacity.

Higher-level platelet-inspired engineering suggests that ideal reversal should avoid indiscriminate hemostasis enhancement and instead use selective enrichment, stimulus responsiveness, and functional gating to relieve inhibition when needed while remaining inert otherwise (Raghunathan et al., 2022). Removable or dissociable reversal and hemostasis integrated systems support the feasibility of shifting from passive neutralization to engineered control (Zeng et al., 2025). Translationally, the focus is quantitative definition of capacity limits, onset and termination kinetics, and deactivatable pathways, together with evidence that the systems neither triggers platelet activation nor causes over-reversal, establishing a releasable balance between lifting inhibition and preserving long-term antithrombotic benefit (Raghunathan et al., 2022; Zeng et al., 2025).

## 7 Translational pathway: CMC and regulation

For platelet-mimicking nanosystems, the bottleneck has shifted from functional feasibility to predictable delivery that is manufacturable, releasable, and regulatable. Small-animal efficacy in hemostasis, inflammation targeting, and immunomodulation is robust, but membrane source and biological variability, scale-up and CQAs, and systemic safety boundaries are often not closed into early design-validation loops, limiting clinical translatability (Luc et al., 2022; Joyce et al., 2024; Safdar et al., 2024; Liu B. et al., 2025). Three questions therefore dominate: the minimal biomimicry tiers and interfaces to retain versus gate, the minimal release package (CQAs with potency and risk surrogates), and upfront validation under worst-case shear, systemic inflammation, repeat dosing, and immune variability (Younis et al., 2022; Joyce et al., 2024).

Membrane source is a first-order CMC constraint. Donor platelet dependence imposes supply and cold-chain or sterility burdens, limited shelf life, donor variability, and poor scalability because membrane coating consumes large platelet numbers (Luc et al., 2022; Safdar et al., 2024). Translation follows a gradient. It begins with donor membranes and progresses bioreactor or stem-cell derived platelets. It then moves toward receptor-engineered or hybrid membranes, and ultimately partially or fully synthetic platelet-inspired interfaces. The goal is controllable composition, scalable manufacturing, and predictable *in vivo* behavior with only the minimal effective interface modules retained (Coradduzza et al., 2025; Liu B. et al., 2025).

CMC must convert research-level authenticity into releasable CQAs and a traceable evidence chain from CQAs and critical process parameters to *in vivo* behavior. In parallel, the computational and ML workflows discussed in Section 5 provide a direct route to operationalize gating behavior as measurable attributes. Specifically, model-derived bounds for trigger threshold, background leakage, and shutdown kinetics can be translated into assayable acceptance windows and incorporated into the release package alongside potency and safety-boundary surrogates. In addition to size and dispersity, surface charge, and membrane coating fraction or stability, interface CQAs should capture membrane protein retention and integrity, epitope availability and receptor orientation, and key residuals (endotoxin, nucleic acids, host proteins) that govern adhesion, recognition, and immunomodulation (Safdar et al., 2024; Coradduzza et al., 2025; Liu B. et al., 2025). Executable release packages should pair these structure and composition CQAs with potency surrogates (shear-dependent adhesion and rolling, lesion binding). They should also include safety-boundary surrogates (e.g., platelet activation/aggregation and baseline coagulation/complement perturbations). This framework helps prevent donor and process heterogeneity from turning into scale-up and regulatory uncertainty (Luc et al., 2022; Coradduzza et al., 2025; Liu B. et al., 2025).

Systemic safety should be framed as verifiable boundaries, not the absence of obvious toxicity. Given the centrality of platelets to coagulation, inflammation, and immunity, function-enhancing designs can shift thrombosis and inflammatory risk, yet current evidence is largely single-dose and small-animal with limited repeat-dosing, long-term, dose-escalation, or human-relevant safety data (Luc et al., 2022; Younis et al., 2022; Coradduzza et al., 2025). Translation therefore requires pre-specified boundary endpoints (platelet activation or aggregation, coagulation-cascade shifts, and confinement to permitted microenvironments or windows) tested under worst-case stress panels spanning high shear, systemic inflammation, repeat dosing, and immune background variability (Luc et al., 2022; Younis et al., 2022).

Complement compatibility and CARPA are unavoidable and immune background dependent. Membrane composition and surface charge, together with protein-corona remodeling, can shift complement activation pathways (Estape Senti et al., 2022; Saxena et al., 2025), and PEGylation is not universally protective because anti-PEG antibodies can amplify complement reactions, destabilize membranes, and increase CARPA risk (Estape Senti et al., 2022). Complement compatibility should therefore be integrated early into design, QC, and release logic alongside functional performance (Estape Senti et al., 2022; Saxena et al., 2025).

Regulation requires a predictable loop from design to manufacturing to *in vivo* behavior. In nanomedicine, translation failures often stem from poor predictability and manufacturing or regulatory alignment rather than lack of efficacy, motivating frameworks such as DELIVER that front-load quality targets and integrate scale-up, stability, safety, and regulatory strategy (Younis et al., 2022; Joyce et al., 2024; Zhang X. et al., 2025). For platelet-mimicking systems, biological components, complex interfaces, and coagulation or immune liabilities heighten uncertainty in attribute definition and CMC review (Luc et al., 2022; Joyce et al., 2024). Clinical potential ultimately rests on a reproducible, releasable balance among biological authenticity, engineering controllability, and systemic safety rather than maximal platelet mimicry.

## 8 Conclusions and outlook

Platelets sit at the immune-coagulation-vascular interface and provide a distinctive entry point for nanomedicine engineering. Over the past decade, platelet functions have been modularized into synthetic platforms. The central value of platelet biomimicry is selective, controllable reconstruction that decouples efficacy from systemic risk within the structure-membrane-function-gating design space.

Across indications, performance gains most often arise from interface state recognition and programmable control at immune-coagulation coupling nodes rather than unconditional procoagulant or aggregation enhancement. Platelet-inspired systems recurrently operate as executors (hemostasis and reversal), navigation gateways (vascular inflammation and ischemia), or gating/buffering nodes (cancer and infection), with success determined by defined, maintainable constraints.

Three principles emerge: functional decoupling of entry from amplification; permission-based activation tied to pathological contexts and time windows; and explicit, releasable risk boundaries grounded in worst-case scenarios (leakage, thresholds, shutdown kinetics, and bounded coagulation or immune perturbation). Translation now hinges on manufacturability, quality consistency, and regulatory predictability, often requiring trade-offs from biological authenticity to definable composition and verifiable controls. Looking ahead, progress will likely bifurcate into minimalist, function-focused systems for predictable delivery, reversal, and buffering, and explicitly gated multimodule systems for high-value indications. In both cases, the benchmark is not apparent platelet-likeness but a manufacturable, verifiable, and regulatable solution to a defined clinical problem.
