# Supplementary material for: Engineering design of platelet-mimicking therapeutic systems: multilevel biomimicry, gating strategies, and translational boundaries
Source: Front Bioeng Biotechnol. 2026 May 13;14:1787749. doi: 10.3389/fbioe.2026.1787749 (PMC13212282; doi:10.3389/fbioe.2026.1787749)
Supplement: Supplementary file 1 [file Image1.pdf]

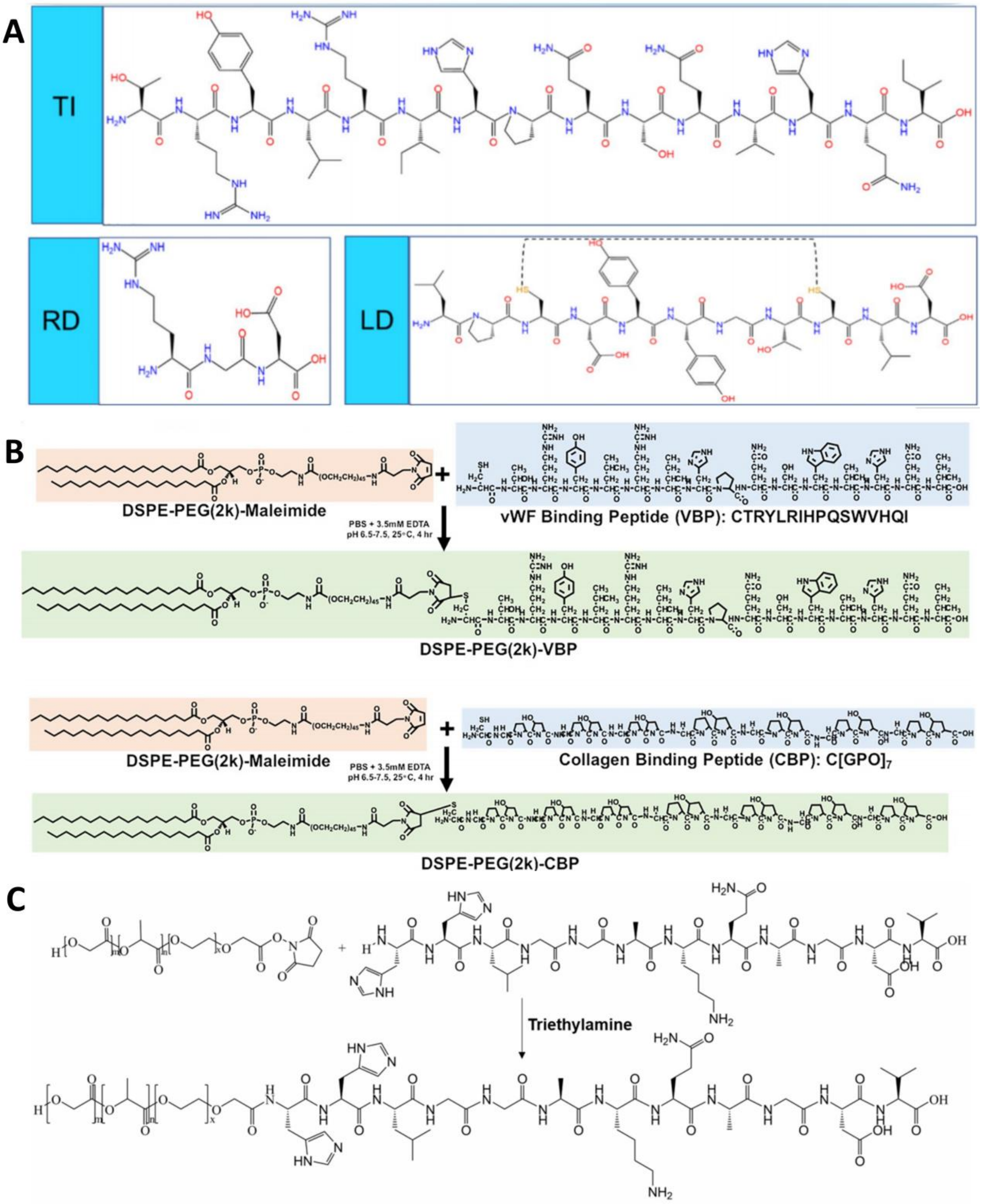

Figure S1. Chemical representations of peptide-based recognition segments and representative conjugation chemistries used in platelet-mimicking systems. (A) Chemical structures of three representative hemostatic peptides: an RGD peptide, a von Willebrand factor-binding peptide, and a fibrin-binding peptide (Shuai et al., 2025). Copyright 2025, J Nanobiotechnology. (B) Thiol-maleimide bioconjugation schemes for synthesizing lipid-poly(ethylene glycol)-von Willebrand factor-binding peptide and lipid-poly(ethylene glycol)-collagen-binding peptide conjugates for surface presentation (Girish et al., 2022). Copyright 2022, ACS Nano. (C) Conjugation scheme for synthesizing a polymer-fibrinogen-mimetic peptide conjugate (PLGA-fibrinogen-mimetic peptide). (Fu et al., 2025). Copyright 2025, Int J Nanomedicine.
